# Nanoscale “Chessboard” Pattern Lamellae in a Supramolecular Perylene-Diimide Polydiacetylene System

**DOI:** 10.3390/molecules30061207

**Published:** 2025-03-07

**Authors:** Ian J. Martin, Francis Kiranka Masese, Kuo-Chih Shih, Mu-Ping Nieh, Rajeswari M. Kasi

**Affiliations:** 1 Department of Chemistry, University of Connecticut, Storrs, CT 06269, USA; imartin9124.im@gmail.com (I.J.M.); fmasese9@gmail.com (F.K.M.); 2 Polymer Program, University of Connecticut, Storrs, CT 06269, USA; juiceshih1230@gmail.com; 3 Institute of Materials Science, University of Connecticut, Storrs, CT 06269, USA; 4 Department of Chemical and Biomolecular Engineering, University of Connecticut, Storrs, CT 06269, USA; 5 Biomedical Engineering, University of Connecticut, Storrs, CT 06269, USA

**Keywords:** perylene diimide, polydiacetylene, PDA, self-assembly, interconnected chessboard pattern

## Abstract

The rational design of ordered chromogenic supramolecular polymeric systems is critical for the advancement of next-generation stimuli-responsive, optical, and semiconducting materials. Previously, we reported the design of a stimuli-responsive, lamellar self-assembled platform composed of an imidazole-appended perylene diimide of varying methylene spacer length (*n* = 3, 4, and 6) and a commercially available diacid-functionalized diacetylene monomer, 10, 12 docosadiynedioic acid, in a 1:1 molar ratio. Herein, we expound on the importance of the composition of the imidazole-appended perylene diimide of varying methylene spacer length (*n* = 3, 4, and 6) and 10, 12 docosadiynedioic acid in the ratio of 2:1 to the supramolecular self-assembly, final morphology, and properties. Topochemical polymerization of the drop-cast films by UV radiation yielded blue-phase polydiacetylene formation, and subsequent thermal treatment of the films produced a thermoresponsive blue-to-red phase transformation. Differential scanning calorimetry (DSC) studies revealed a dual dependence of the methylene spacer length and stimuli treatment (UV and/or heat) on the thermal transitions of the films. Furthermore, small-angle X-ray scattering (SAXS) and wide-angle X-ray scattering (WAXS) showed well-defined hierarchical semiconducting nanostructures with interconnected “chessboard”-patterned lamellar stacking. Upon doping with an ionic liquid, the 2:1 platform showed higher ionic conductivity than the previous 1:1 one. The results presented here illustrate the importance of the composition and architecture to the ionic domain connectivity and ionic conductivity, which will have far-reaching implications for the rational design of semiconducting polymers for energy applications including fuel cells, batteries, ion-exchange membranes, and mixed ionic conductors.

## 1. Introduction

Polydiacetylenes (PDAs) have emerged as a useful class of stimuli-responsive polymeric materials [1,2,3,4]. They have garnered much interest for their potential in a wide range of colorimetric applications based on their known chromogenic transitions upon polymerization and stimuli-induced “blue→red phase” transition [5,6,7,8]. The pioneering work on PDAs began with Wegner in the 1960s with his investigations into the 1, 4 solid-state topochemical polymerization of hexadiyne-diol [9,10,11]. Since then, PDA research has grown substantially, establishing itself in such potential applications as biomedical devices [12], damage-sensing materials [13], optoelectronic devices [14], and chemosensors [15,16]. There are strict geometrical requirements for neighboring DA monomers to polymerize [17]. To meet DA monomer topochemical prerequisites, a variety of functionalities are attached to the monomer, including, but not limited to, hydrogen-bonding moieties [18,19,20], π-π stacking groups [21,22,23], and metal-coordinated motifs [24,25,26,27,28,29].

A variety of supramolecular PDAs with an array of architectures have been developed for sensing applications [30,31,32,33]. In many cases, the formation of supramolecular PDA platforms is facilitated by hydrogen-bonding groups, π-π stacking moieties, or a combination of the two [34,35]. Due to the strong affinity for perylene diimides (PDIs) to form into columnar objects by π-π stacking [36,37,38,39] and their known conductive properties [40,41], they have been utilized as ideal candidates for novel, conductive PDA systems. Both covalent and supramolecular PDI-PDA systems have been studied for their potential use in colorimetric and conductive applications [9,42,43,44,45,46]. Recently, our group developed a series of novel PDI-PDA templated polymeric structures capable of reversible multichromic and multiresponsive pathways [47]. The supramolecular templated formation relied on the imidazole–acid interactions of commercially available 10, 12 pentacosadiynoic acid (PCDA) and imidazole-appended PDI templates of various spacer length by hydrogen bonding. UV irradiation of solution-cast films yielded films capable of solvato- and thermochromic reversibility switching from the purple and red phases of PDA. The phase reversibility of these systems may be a result of the hydrogen bonding of the PDI–imidazole and carboxylic acid groups lowering the difference in conformational energy between the purple and red phases. X-ray studies including SAXS and WAXS studies revealed an arrangement of hydrogen-bonded PDI-PCDA cylinders amongst poly(PCDA)-rich domains composed of red and blue phases. These templated supramolecular polymeric PDI-PDA systems may have potential applications in sensors and conductive materials [48].

Due to the semicrystalline properties of PDAs and their derivatives, it is extremely challenging to synthesize architectures that can self-assemble into interconnected, gyroid-like or bicontinuous morphologies [18,49,50,51]. These morphologies would be immensely interesting to address if the transport and diffusion of ions and charged molecules are higher or lower than those of randomly oriented or aligned lamellar or cylindrical structures, which has been a major point of contention in the block copolymer field [51,52,53]. Furthermore, the transport of penetrants through nanoscale-oriented and disordered morphologies has been of interest for electrochemical devices [53], fuel cells [54], separation membranes [55], and batteries [56].

In this work, we designed a general strategy using compositions with different architectures as a tool to synthesize PDA-PDI systems that self-assembled into interconnected morphologies. Building on this framework of supramolecular PDI-PDA systems, this work seeks to investigate a supramolecular PDI-PDA polymer system composed of a central diacid DA monomer, commercially available 10, 12 docosadiynedioic acid (**DCDDA**), hydrogen-bonded to a series of two imidazole-appended PDI molecules, where the imidazole group is attached to PDI by a variable methylene spacer length, *n* (*n* = 3, 4, and 6) (**PDI-mono**(***n*-imz**)). Upon complexation in solution, supramolecular formation occurs in a 2:1 molar ratio of **PDI-mono**(***n*-imz**) and **DCDDA** (**2:1 PDI-mono**(***n*-imz**)**/DCDDA**) through intermolecular π-π stacking of neighboring PDI molecules and imidazole–acid hydrogen bonding. This method allows for a modular approach to supramolecular PDI-PDCDDA systems where the properties of the materials can be easily tailored by substitution of the diacid-functionalized DA monomer or imidazole-appended PDI molecule prior to polymerization. Morphological studies before and after UV polymerization of the supramolecular systems displayed hierarchical ordering on multiple length scales and the effects of crystallinity on varying the methylene spacer length. Additionally, thermal studies revealed several trends in relation to varying the methylene spacer lengths of the PDI molecules in the supramolecular systems. Finally, the chromatic transitions were investigated and showed various pathways to achieve blue- and red-phase transitions in the PDA polymer backbone, where these 2:1 systems were compared and contrasted with the corresponding 1:1 architectures and used as model systems to investigate ionic conductivity in these films compared with lamellar-domain-forming PDA-PDI structures.

## 2. Results and Discussion

**Figure 1:**
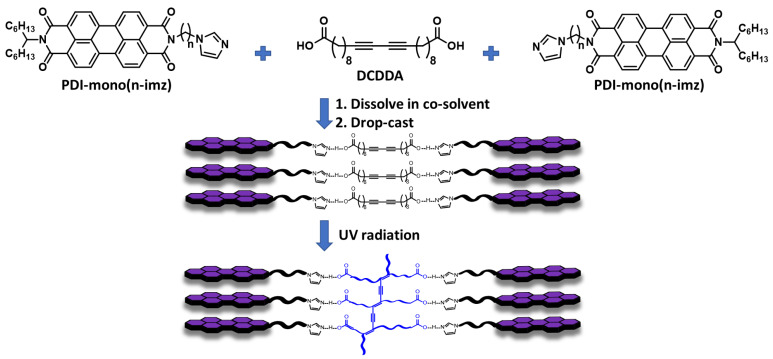
Schematic illustration of the supramolecular formation of **DCDDA** and **PDI-mono***(**n-*****imz**) to form **2:1 PDI-mono**(***n*-imz**)**/DCDDA** and their subsequent polymerization to the blue phase by 254 nm UV radiation.

The PDI molecules used in this study (**PDI-mono**(***n*-imz**)) were synthesized according to the previous literature [47,57,58] and were composed of unsymmetrical PDI functionalized with a variable methylene spacer length, *n* (*n* = # of methylene units: 3, 4, or 6), attached to a basic imidazole headgroup on one end and a dialkyl-solubilizing moiety on the other end (Figure 1). The diacid DA monomer used in this work, 10, 12 docosadiynedioic acid (**DCDDA**), is commercially available, and it was purified to remove any polymerized byproducts. The **PDI-mono**(***n*-imz**) molecule and purified **DCDDA** monomer were dissolved in a 2:1 molar ratio (**2:1 PDI-mono**(***n*-imz**)**/DCDDA**) at 2 wt % in anhydrous THF and stirred overnight in a sealed, dark container at room temperature. The resulting solutions were drop-cast onto dry quartz substrates at room temperature with no ambient light and allowed to dry completely to prepare supramolecular hydrogen-bonded structures. Samples that were examined prior to photopolymerization were analyzed upon drying, and a 254 nm UV source (7.6 mW cm^−2^, 10 min) was used to polymerize the drop-cast films prior to further analysis. Films that underwent stimuli treatment are denoted by “UV mx Heat mx”, where “UV” = 254 nm exposure for 10 min at room temperature and “Heat” = 150 °C for 1 h in air. “UV mx Heat mx” refers to “m” # of treatments by “UV” and/or “Heat”.

### 2.1. Supramolecular Complexation

**Figure 2:**
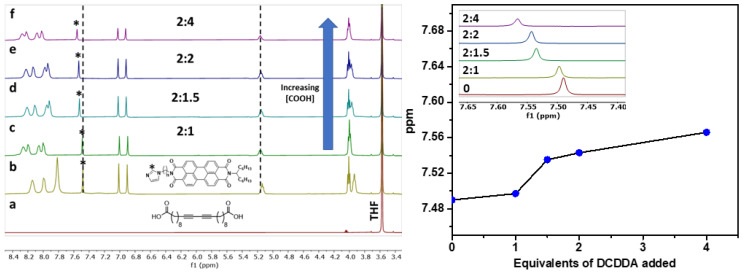
(**Left**) ^1^H NMR spectrum overlay of (a) **DCDDA**, (b) **PDI-mono**(**6-imz**), (c) 2:1 **PDI-mono**(**6-imz**)**/DCDDA**, (d) 2:1.5 **PDI-mono**(**6-imz**)**/DCDDA**, (e) 2:2 **PDI-mono**(**6-imz**)**/DCDDA**, and (f) 2:4 **PDI-mono**(**6-imz**)**/DCDDA** in THF-d_8_ at 25 °C. The imidazole proton that is denoted by an asterisk (*) shifts from 7.48 ppm to 7.56 ppm, and the tertiary proton chemical shift at 5.18 ppm does not shift upon hydrogen bonding. (**Right**) Chemical shift change of PDI aromatic imidazole proton (labeled with * in the ^1^H NMR spectrum overlay) upon the addition of increasing amounts of **DCDDA**. Points represent experimental data. The inset shows the ^1^H NMR spectrum overlay of the aromatic imidazole proton peak from 7.64 ppm to 7.40 ppm of **PDI-mono**(**6-imz**)**:DCDDA** with increasing amounts of **DCDDA**.

The supramolecular **PDI-mono**(**6-imz**)**/DCDDA** structures formed in solution were analyzed by ^1^H NMR spectroscopy in tetrahydrofuran-d_8_ (THF-d_8_) at room temperature at increasing concentrations of **DCDDA** (Figure 2). The aromatic proton in the imidazole ring (labeled with *) successively shifts downfield from 7.49 ppm to 7.57 ppm upon increasing equivalents of **DCDDA**. This is a result of the hydrogen bonding between the carboxylic acid groups of **DCDDA** and the imidazole head groups of **PDI-mono**(**6-imz**). Additionally, the proton shifts associated with the PDI aromatic core broaden, indicating supramolecular formation, in agreement with the previous literature [58]. The titration profile obtained with increasing amounts of **DCDDA** added shows a critical transition when approximately 2 equiv of **PDI-mono**(**6-imz**) is used for 1.25 equiv of the diacid, which deviates from the expected 2:1 supramolecular structure. This deviation may be a result of the limited solubility of **PDI-mono**(**6-imz**) in THF-d_8_ in comparison to **DCDDA** at 2 wt%. This will lead to a lower concentration of **PDI-mono**(**6-imz**)**:DCDDA** in solution, which may be the reason for the greater-than-expected experimental equivalence point. This may result in the drop-cast films not adopting the expected 2:1 supramolecular structure, as not all of the **DCDDA** carboxylic acid units complex with the **PDI-mono**(***n*-imz**) molecules. This phenomenon is consistently observed for shorter methylene spacer lengths of *n* = 3 and 4 (Figures S1 and S2, respectively) as well.

**Figure 3:**
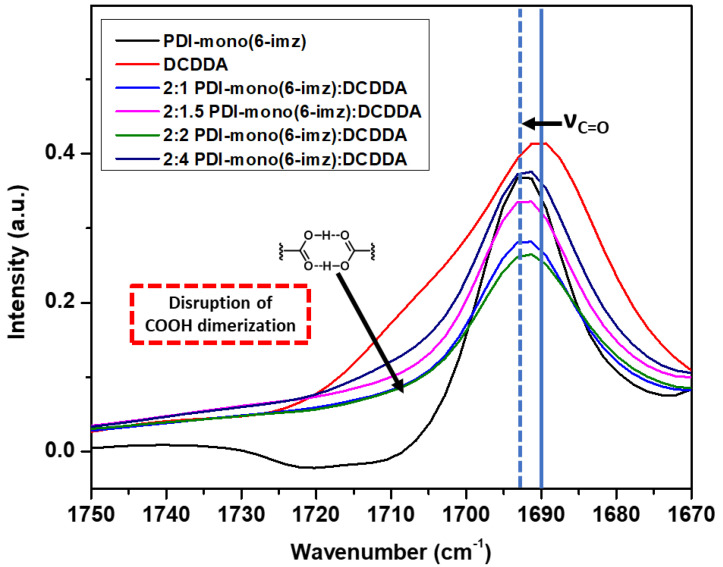
FTIR–ATR spectrum overlay of **PDI-mono**(**6-imz**) (black line), **DCDDA** (red line), 2:1 **PDI-mono**(**6-imz**)**/DCDDA** (blue line), 2:1.5 **PDI-mono**(**6-imz**)**/DCDDA** (pink line), 2:2 **PDI-mono**(**6-imz**)**/DCDDA** (green line), and 2:4 **PDI-mono**(**6-imz**)**/DCDDA** (purple line) powder at 25 °C. The downward arrow at ~1710 cm^−1^ shows the decrease in free COOH absorbance after imidazole–acid complexation. The solid–to–dashed blue line shift from 1690 cm^−1^ to 1693 cm^−1^ represents the disruption of dimerized COOH moieties after imidazole–acid complexation.

Upon drop-cast film formation, the supramolecular structures of **PDI-mono**(**6-imz**)**:DCDDA** were shown to maintain imidazole–acid complexation in the solid state according to Fourier transform infrared (FTIR) spectroscopy analysis (Figure 3). Similar acid-appended DA monomers organize in the solid state by carboxylic acid dimerization [59]; therefore, the addition of a molecule with a basic binding site, such as imidazole, should disrupt the dimerization phenomenon [60,61]. This is seen when comparing the absorbance from 1725 cm^−1^ to 1700 cm^−1^, which represents carboxylic acid dimerization stretching, of pure **DCDDA** (red line) and **2:1 PDI-mono**(**6-imz**)**:DCDDA** (blue line), where there is clearly a large decrease in the signal intensity. Furthermore, there is a gradual increase in the intensity of this band as the concentration of **DCDDA** is increased. Additionally, the solid blue vertical line shifting at 1690 cm^−1^ to 1693 cm^−1^ (dashed blue vertical line) represents the change in C=O stretching when carboxylic acid dimerization is disturbed. This phenomenon is also consistent with complexes composed of shorter methylene spacer lengths of *n* = 3 and 4 (Figures S3 and S4, respectively).

### 2.2. Chromatic Transitions

**Figure 4:**
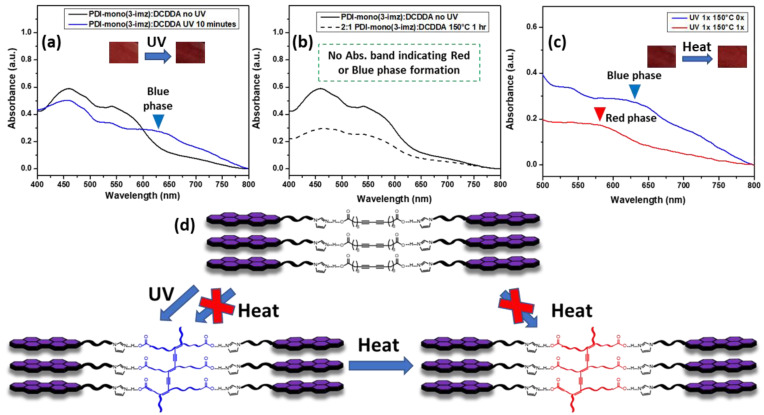
UV–Vis spectra of **2:1 PDI-mono**(**3-imz**)**/DCDDA** drop-cast films indicating (**a**) blue-phase PDA formation after UV irradiation, (**b**) no PDA formation after thermal treatment at 150 °C for 1 h, (**c**) blue-to-red-phase PDA transformation upon after thermal treatment at 150 °C for 1 h, and (**d**) stimuli-specific chromatic transition diagram of **2:1 PDI-mono**(**3-imz**)**/DCDDA** supramolecular system. The insets in (**a**,**d**) show photographs of **2:1 PDI-mono**(**3-imz**)**/DCDDA** drop-cast films before/after UV and after UV/after UV and heating, respectively.

The chromatic/stimuli-responsive phase behavior of the 2:1 **PDI-mono**(**3-imz**)**/DCDDA** drop-cast film on soda-lime (microscope) glass is shown in Figure 4. Prior to UV treatment, the UV–Vis spectrum overlay in Figure 4a shows only a contribution of **PDI-mono**(**3-imz**). However, after UV exposure, an absorption band at ~625 nm appears, which can be attributed to polymerized **DCDDA** (labeled **PDCDDA**) (Figure S5). Interestingly, thermal treatment of the 2:1 **PDI-mono**(**3-imz**)**/DCDDA** drop-cast film at 150 °C for one hour prior to UV exposure did not show a new absorption band indicating red- or blue-phase formation (Figure 4b). Thermal treatment at 150 °C was chosen because it is above the T_m_ of **DCDDA**, as determined by differential scanning calorimetry (DSC) (Figure S9). This contrasts with the thermal treatment of the **DCDDA** powder (red line, Figure S5), where there is an increase in the absorption intensity at ~575 nm, which is typically where the red-phase PDA absorption band appears [62,63,64,65]. This discrepancy may be a result of the **PDI-mono**(**3-imz**) molecule absorption band overlapping with this in the UV–Vis spectrum, or a result of the PDI molecule inhibiting or decreasing the formation of a red phase in the PDCDDA backbone. In Figure 4c, it is shown that red-phase formation may occur by heating the blue-phase 2:1 **PDI-mono**(**3-imz**)**/DCDDA** drop-cast film to 150 °C for one hour, indicated by the disappearance of the blue-phase absorption band (blue line) and the appearance of a red-phase absorption band (red line). From this, a phase diagram was constructed to determine routes to achieve various PDCDDA phases in the 2:1 **PDI-mono**(**3-imz**)**/DCDDA** drop-cast films (Figure 4d) based on the UV–Vis absorption data. It can be concluded that PDCDDA blue-phase formation occurs due to UV irradiation and not heat treatment. It can also be inferred that red-phase formation occurs through forming blue-phase PDA first by UV radiation and then by heat treatment. Interestingly, this occurs at longer methylene spacer lengths of *n* = 4 and 6 (Figures S6 and S7, respectively), which suggests that the effect of the methylene spacer length is minimal when transitioning between the blue and red PDCDDA phases.

Thermal analysis of the DCDDA and 2:1 PDI-mono(n-imz)/DCDDA drop-cast films before and after UV irradiation was performed using thermogravimetric analysis (TGA) (Supporting Information, Figure S8, Table S1). The thermal stability of DCDDA was observed to exhibit a distinctive degradation behavior after UV irradiation, possibly resulting in an intermolecular 1,4-addition reaction to yield highly crosslinked networks [66,67]. Comparatively, the 2:1 PDI-mono(4-imz)/PCDA drop-cast films were more thermally stable than the 2:1 PDI-mono(3-imz)/PCDA films (Figure S8). This observation is attributed to the flexible alkyl spacers, which facilitate the PDI π-π interactions, thus forming ordered and stable assemblies.

The thermal transitions of these films before and after exposure to various stimuli were investigated by differential scanning calorimetry (DSC) after being subjected to a series of heating and cooling cycles at a ramp rate of 5 °C min^−1^ (Supporting Information, Figures S9–S12, Table S2). Similar to our initial report [47], all the drop-cast films exhibited a characteristic sharp melting transition of DCDDA but at higher temperatures in the second heating cycle. Upon cooling, the **2:1 PDI-mono**(**n-imz**)**/DCDDA** drop-cast sharp crystalline transition temperature values of **DCDDA** (Tc,DCDDA) were observed. This suggests that at lower temperature, the disordered **2:1 PDI-mono**(**n-imz**)**/DCDDA** supramolecular molecules lower the decoupling of the DCDDA domains from the arranged PDI templates, which undergo rearrangement to form well-packed crystalline structures [47].

### 2.3. Morphological Properties

**Figure 5:**
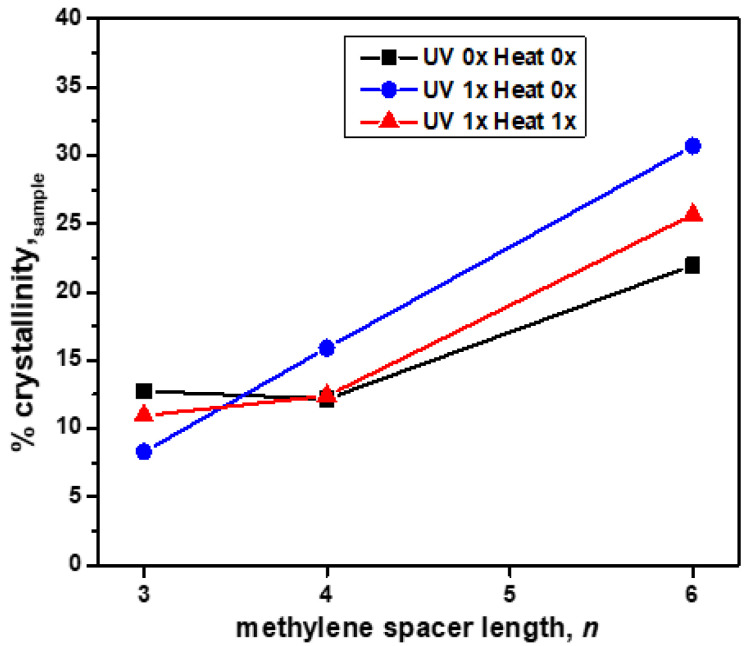
The % crystallinity of samples of **2:1 PDI-mono**(***n*-imz**)**/DCDDA** drop-cast films calculated from WAXS (% crystallinity,_sample_) as a function of the methylene spacer length, *n* (where *n* = 3, 4, or 6), where black squares, blue circles, and red triangles represent the stimuli treatment provided to the drop-cast films (UV mx Heat mx).

Wide-angle X-ray scattering (WAXS) was used to study the effects of the methylene spacer length, *n* (where *n* = 3, 4, or 6), on crystallinity in the 2:1 **PDI-mono**(***n*-imz**)**/DCDDA** drop-cast films prior to **DCDDA** polymerization, after blue-phase formation by UV exposure and subsequent red-phase formation by heat treatment. The WAXS spectrum overlays of the 2:1 **PDI-mono**(***n*-imz**)**/DCDDA** drop-cast films after various stimuli treatments (Figures S13–S16) display peaks indicative of **DCDDA**-rich regions (d_1_, d_4_) and intermolecular π-π stacking of **DCDDA** (d_6_) and **PDI-mono**(***n*-imz**) (d_7_). This, in conjunction with the FTIR data, shows that a combination of hydrogen bonding and π-π stacking aids in DA alignment in the supramolecular structures. The % crystallinity of the 2:1 **PDI-mono**(***n*-imz**)**/DCDDA** drop-cast films after various stimuli treatments was measured by comparing the crystalline to amorphous regions (2θ = 4–30°) by WAXS and plotted as a function of the methylene spacer length, *n*, in the supramolecular structures (Figure 5). The % crystallinity of the sample increases with increasing methylene spacer length, potentially as a result of the enhanced packing of PDI/PDCDDA cylinder domains with greater methylene spacer flexibility (peak d_2_, Figures S14–S16). Additionally, when *n* = 4 and 6, the crystallinity of the supramolecular structures is enhanced upon UV treatment, followed by a reduction upon heating. This may be a result of the longer, more flexible methylene spacer lengths attached to **PDI-mono**(***n*-imz**) allowing for a higher degree of blue-phase formation of the **PDCDDA** domains as compared to when *n* = 3. Upon heating and red-phase formation, there is a reduction in the sample crystallinity, which may result from the **PDCDDA** backbone relaxing by undergoing a rotation about the C-C single bond [56]. This acts to disrupt the extent of crystallinity of the **PDCDDA** domains. Interestingly, the crystallinity slightly increases after heating when *n* = 3, which highlights how the increased methylene spacer length in **PDI-mono**(***n*-imz**) may allow for a greater extent of blue-phase formation due to a higher degree of decoupling of the bulky, aromatic substituent from the **PDCDDA** backbone.

**Figure 6:**
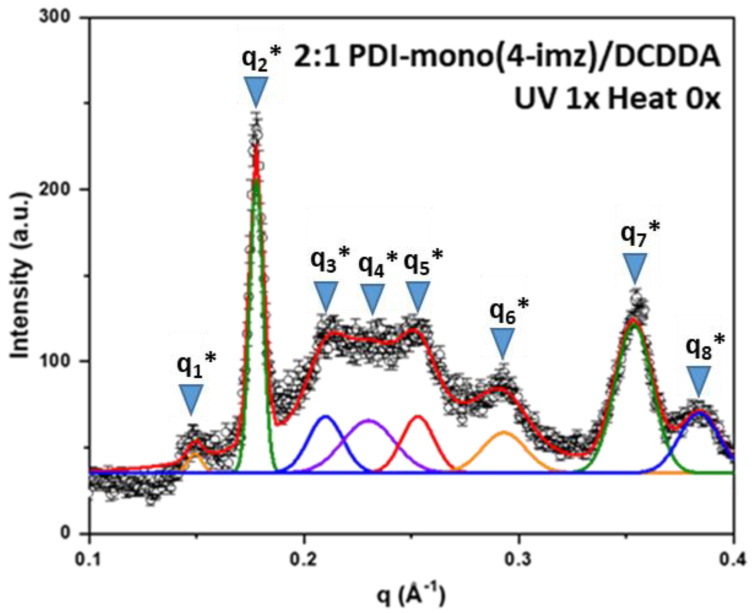
The 1−D SAXS pattern of 2:1 **PDI-mono**(**4-imz**)**/DCDDA** after UV exposure (“UV” = 254 nm exposure for 10 min at room temperature, and “Heat” = 150 °C for 1 h). SAXS peak assignments (q_n_*) can be found in Table 1. Peak positions were determined using the fitting function in Origin.

**Figure 7:**
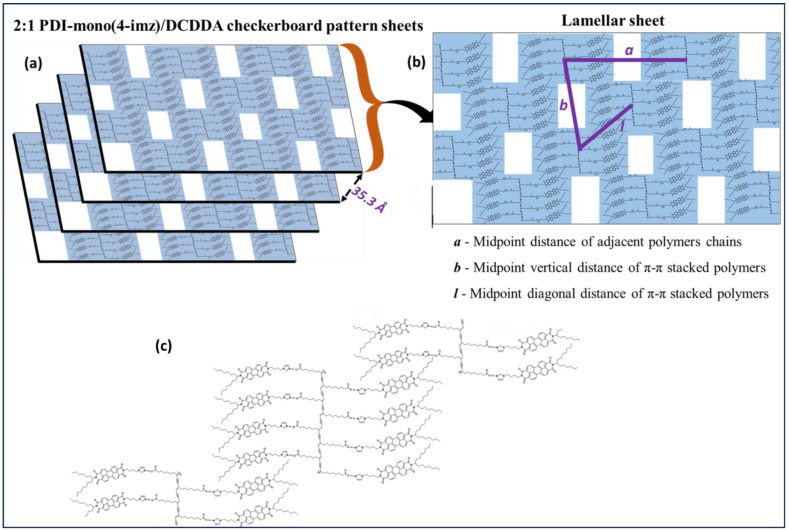
Schematic illustration of (**a**) self-assembled 2:1 **PDI-mono**(**4-imz**)**/PDCDDA** into “chessboard” pattern sheets after UV exposure (“UV” = 254 nm exposure for 10 min at room temperature, and “Heat” = 150 °C for 1 h.). (**b**) Single lamellar sheet, and (**c**) head-with-head and head-with-tail configurations of the polymer.

Small-angle X-ray scattering (SAXS) was used to study the various structures within the 2:1 **PDI-mono**(***n*-imz**)**/DCDDA** drop-cast films. Figure 6 shows a representative 1D SAXS pattern of 2:1 **PDI-mono**(**4-imz**)**/DCDDA** after UV exposure for 10 min. The curve is best fit by several Gaussian peaks to resolve individual peak positions, q_n_^*^, and their values are listed in Table 1. Note that the peak positions cannot be interpreted by a known crystal structure. Herein, we propose stacking “chessboard”-patterned lamellae [as shown in the scheme of Figure 7a] to describe the SAXS pattern presented in Figure 6. The rationale of this proposed model originates from the combined effects of polymerization and π-π stacking. The first peak, q_1_* = 0.149 Å^−1^, corresponds to a d-spacing of $\frac{2\pi }{q_{1}^{*}}$ (=42.1 Å), which is approximately the molecular length of nearly stretched **PDI-mono**(**3-imz**)**/DCDDA**. It should be noted that the π-π stacking at the end of the side chains promotes two potential **PDI-mono**(**3-imz**)**/DCDDA** aggregating configurations before the polymerization takes place, i.e., head-with-head (**PDI-mono**(**3-imz**)**/DCDDA** stacking with each other) and head-with-tail (**PDI-mono**(**3-imz**)**/DCDDA** connected by ends) (Figure 7b). Polymerization could chemically connect the head-with-head aggregates into a near-rectangular lamella (hereafter referred to as a “patch”), with its four corners remaining “active” for the head-with-tail п-п stacking as the “glue” points for other patches. As a result, all the corner-connected patches will form chessboard-like lamellae [Figure 7c]. We define the long axis of **PDI-mono**(**3-imz**)**/DCDDA** and the polymerization direction as the “*a*” and “*b*” axes, respectively, which form a 2D lattice crystal on the lamella. It should be noted that the “*a*” and “*b*” axes are not necessarily perpendicular to each other. The repeat spacing along the *a* axis is dictated by the molecular length of **PDI-mono**(**3-imz**)**/DCDDA**; hence, q_1_* is assigned to the first-order diffraction peak along the *a* axis, i.e., (1,0).

The second peak, q_2_* (=0.178 Å^−1^), and the seventh peak, q_7_* (=0.353 Å^−1^), are much sharper than the others, suggestive of different origins. The fact that q_7_* is around 2q_2_* indicates that q_7_* is the second-order harmonic of q_2_*. The well-defined corresponding d-spacing of 35.3 Å (=$\frac{2\pi }{q_{2}^{*}}$) most likely originates from the interlamellar distance.

The third diffraction peak, q_3_*, at 0.210 Å^−1^ is assigned to the first-order Bragg peak along the *b* axis, i.e., (0,1), corresponding to a repeat spacing of $\frac{2\pi }{q_{3}^{*}}$ (=29.9 Å) [Figure 7c]. The uniformity of the d-spacing along the *b* axis was not anticipated and is well understood since it is presumably controlled by entropy. Because of the “chessboard” configuration, we expect that there is a regular spacing along the diagonal direction [*l* in Figure 7c] between the *a* and *b* axes, which is $\frac{\sqrt{a^{2}+b^{2}}}{2}$ (~25.8 Å) under the approximation of nearly orthogonal *a* and *b* axes, leading to a Bragg reflection at 0.243 Å^−1^, close to peak q_4_* (~0.240 Å^−1^), which is evidence of the “chessboard” configuration. The fifth peak, q_5_* (~0.253 Å^−1^) $\sim \sqrt{{q_{1}^{*}}^{2}+{q_{3}^{*}}^{2}}\left(=0.25\;{Å}^{-1}\right)$ (if the *a* and *b* axes are nearly orthogonal), can be interpreted as the Bragg reflection from the (1,1) plane. The sixth peak, q_6_*~2q_1_*, is indicative of the second-order harmonic along the *a* axis, consistent with the WAXS pattern, where a higher-order (the fourth) Bragg reflection is found at q = 0.606 Å^−1^ for the **DCDDA** sample (Figure S13). Finally, the reflection of q_8_^*^ is associated with **PDI-mono**(**4-imz**) [50], which is observed in PDI-rich regions within the supramolecular structures. The fact that the SAXS/WAXS data show a similar pattern to that from a reported intercalated layered structure [68] further validates the structural interpretation. Scanning electron micrographs of the samples provided inconclusive results and are not presented here.

Comparing the SAXS data (q_n_ represents “UV 0x Heat 0x”, q_n_^*^ represents “UV 1x Heat 0x”, and q_n_^**^ represents “UV 1x Heat 1x” (Å^−1^)) and the corresponding d-values (d_n_, d_n_^*^, d_n_^**^ (Å)) of the DCDDA powder before and after UV exposure (Figure S22, Table S10) to the 2:1 **PDI-mono**(***n*-imz**)**/DCDDA** drop-cast films (Figures S19–S21, Tables S7–S9) shows that the characteristic peak in **DCDDA** (before and after UV exposure) is present in the supramolecular structures that have spacer lengths of *n* = 4 and 6, both before and after UV exposure. This characteristic peak was only observed in the **PDI-mono**(***n*-imz**)**/DCDDA** polymer with *n* = 3, after the film was exposed to UV, which enhanced the large-scale regularity of the supramolecular structure. This indicates that there are **DCDDA**-rich regions in the supramolecular structures. This may be a result of incomplete complexation of **DCDDA** acid end groups to PDI–imidazole moieties. This phenomenon is in agreement with the ^1^H NMR titration experiment (Figure 2), where the equivalent point was found to be 1.25 moles of **DCDDA:** 2 moles of **PDI-mono**(**6-imz**), in comparison to the theoretical 1:2 **DCDDA:PDI-mono**(**6-imz**) ratio. Since the 2:1 **PDI-mono**(***n*-imz**)**:DCDDA** drop-cast films were prepared at the same concentration as in the ^1^H NMR experiment (2 wt%), it is expected that the films will have a similar ratio, which may lead to **DCDDA**-rich regions because of incomplete acid complexation in solution.

Additionally, exposure of all spacer lengths to UV enhances the large-scale space regularity, where the length of the spacer is observed to play a very important role, resulting in the appearance of the q_4_* peak after UV exposure (Figures S19–S21). Subsequent thermal exposure of the films at 150 °C for one hour leads to peak broadening (q_2_^**^ and q_7_^**^ in all spacer lengths) and the disruption of the 4. q4* q4* distance between two adjacent stacked PDI/PDCDDA polymers (distance L), leading to the disappearance of q_4_^*^ in all spacer lengths. This may be a result of thermal exposure disrupting the order and/or the thermochromic blue→red phase transition changing the domain sizes within the supramolecular structures.

### 2.4. Ionic Conductivity of the 2:1 PDI-Mono(4-imz)/PDCDDA and 1:1 Drop Cast Films

The ionic conductivity of the **2:1 PDI-mono***(***4*-*imz**)**/PDCDDA** polymer was assessed using an Ossila Four-Point Probe System, and the values were compared with those from **1:1 PDI-mono**(**4-imz**)**/PDCDDA.** Both polymers were doped with 0.04 mL of 1-Butyl-3-methylimidazolium acetate to enhance their ionic conductivity. The results show that the **2:1 PDI-mono***(***4*-*imz**)**/PDCDDA** polymer chessboard pattern (Figure 7) exhibited a two-fold enhancement in ionic conductivity compared to the **1:1 PDI-mono**(**4-imz**)**/PDCDDA** lamellar structures formed upon evaporation-induced self-assembly (Table 2). These well-defined chessboard patterns facilitate the close π-π bond stacking interactions, well suited for transferring electrons from molecule to molecule, and therefore play a key role in organic conductors [69]. Note that the PDI films were spiked with 1-butyl-3-methylimidazolium acetate, an ionic liquid with an ionic conductivity value of 1.44 mScm^−1^ [70], to enhance the conductivity of the films. These data values agree with the SAXS data, where the diffraction pattern attributed to the π-π stacking is sharp and well defined. When the samples were subjected to UV exposure, higher conductivity values were observed in both cases, thus indicating an improvement in the well-defined chessboard patterns (Tables S11 and S12).

Differential scanning calorimetry (DSC) and thermogravimetric analysis (TGA) were used to analyze the thermal properties of the materials. After **DCDDA** complexation with **PDI-mono**(***n*-imz**), the decomposition temperatures (T_d_) decreased as compared to pure **DCDDA**, as analyzed by TGA (Figure S8). DSC analysis revealed varying effects of the methylene spacer length, *n*, and stimuli treatment method on the 2:1 **PDI-mono**(***n*-imz**)**/DCDDA** drop-cast films and **DCDDA** powder in the second heating cycle (Figures S9–S12). The melting transition temperatures (T_m,DCDDA_), enthalpies of melting transition (∆H_m,DCDDA_), crystallization transition temperatures (T_c,DCDDA_), and enthalpies of crystallization transition (∆H_c,DCDDA_) of the **DCDDA** regions as a function of the methylene spacer length, *n*, and stimuli treatment can be found in Table S2. For 2:1 **PDI-mono**(**3-imz**)**/DCDDA** (Figure S10), all samples have a T_m,DCDDA_ on the heating cycle and all have a T_c,DCDDA_ on the cooling cycle, besides the sample with no UV or heat treatment (dashed black line). This potentially indicates that UV/thermal exposure allows for greater packing in the supramolecular structures at smaller methylene spacer lengths, whereas the sample treated with neither stimulus has a lower degree of packing. Interestingly, at a longer methylene spacer length of *n* = 6 (Figure S12), all samples have a T_m,DCDDA_ and T_c,DCDDA_ regardless of UV or heat exposure. This may be a result of the extended spacer length, which may decouple the bulky aromatic PDI core from the PDCDDA backbone and/or DA crystalline regions, allowing for greater packing. This is in agreement with the trend of increasing methylene spacer length and subsequent increased crystallinity in the supramolecular structures found in the WAXS analysis.

Figure 7 displays a schematic illustration detailing the phase transitions observed in **2:1 PDI-mono**(***n*-imz**)**/DCDDA** drop-cast films based on SAXS measurements after various stimuli treatments (Figures S19–S21, Tables S7–S9). Prior to any stimuli treatments (“UV 0 × Heat 0×”), the supramolecular structures at all methylene spacer lengths *n* show some uniform ordering that is most likely a result of hydrogen bonding due to imidazole–acid interactions, π-π stacking of adjacent PDI molecules, and, to a lesser extent, van der Waals attractions of long alkyl chains. Upon UV exposure for 10 min (“UV 1x Heat 0x”), blue-phase formation by covalent linkages of neighboring **DCDDA** monomers increases the uniform ordering within the films. Thermal treatment of the blue-phase films (“UV 1x Heat 1x”) for 60 min at 150 °C results in red-phase PDCDDA formation, which in turn disrupts the uniform ordering within the supramolecular structures.

A comparison of morphologies to our previous work [47] on supramolecular templated 1:1 **PDI-mono**(***n*-imz**)**/PCDA** drop-cast films shows the similarities of PDI and **DCDDA** π-π stacking and acid–imidazole hydrogen bonding from ^1^H NMR, FTIR, and WAXS analyses, and similarities in the supramolecular structure formed are seen in the SAXS data. The 1:1 structure formed PPCDA-rich regions that alternated with cylindrical PDI-PPCDA hydrogen-bonded columnar stacks. The SAXS analysis of the 1:1 **mono**(**4-imz**)**/PCDA** drop-cast films also shows evidence of PPCDA-rich regions that somewhat alternate to an extent with the PDI cylindrical structures. Interestingly, the comparison of the DSC data does not show the same thermal transition trends of crystallinity. The 1:1 templated structure only exhibits a T_c_ at shorter methylene spacer lengths, whereas the 2:1 structure displays a T_c_ at the three- and six-spacer lengths (Figures S10 and S12). This may be a result of the “di-complexation” of **DCDDA** by the **PDI-mono**(***n*-imz**) molecules providing less mobility, which causes crystallization to occur at longer methylene spacer lengths. Furthermore, the drop-cast films did not exhibit purple-phase PDCDDA, which is understood as a coexistence of the red and blue phases. Instead, the blue phase and red phase could be selectively triggered through distinct stimuli pathways, which enables applications in sensing and stimuli-responsive functional materials.

## 3. Materials

N-(1-hexylheptyl)-N′-((1-imidazole)propyl)perylene-3,4,9,19-tetracarboxyl-3,4-anhydride-9,10-bisimide (**PDI-mono**(**3-imz**) [47], N-(1-hexylheptyl)-N′-((1-imidazole)butyl)perylene-3,4,9,19-tetracarboxyl-3,4-anhydride-9,10-bisimide (**PDI-mono**(**4-imz**) [47], and N-(1-hexylheptyl)-N′-((1-imidazole)hexyl)perylene-3,4,9,19-tetracarboxyl-3,4-anhydride-9,10-bisimide (**PDI-mono**(**6-imz**)) [58] were synthesized following previously reported procedures. 1-(4-aminobutyl)imidazole (**2**) was synthesized following modified procedures [71,72]. Anhydrous dichloromethane (CH_2_Cl_2_) (99.8%) was purchased from Acros Organics and used without further purification. 10,12 docosadiynedioic acid (DCDDA) (97%) was purchased from Alfa Aesar, dissolved in anhydrous THF, and passed through a 0.45 μm GPC filter to remove polymerized byproducts prior to use. Tetrahydrofuran-d_8_ (D, 99.5%) was purchased from Cambridge Isotope Laboratories Inc. (Tewksbury, MA 01876, USA) and used without further purification.

### 3.1. Materials Synthesis

#### 3.1.1. Representative Drop-Cast Film Preparation

A clean, dry 15 mL plastic amber-colored bottle was tared with the cap on and a small magnetic stir bar inside. **PDI-mono**(**3-imz**) (2.0 equiv.) was added to the plastic bottle, followed by a pure DCDDA stock solution (20 mg/mL, 1.0 equiv.) and anhydrous CHCl_3_ in a sunlight/UV-free room. The resulting 2 wt% **2:1 PDI-mono**(**3-imz**)**/DCDDA** solution was allowed to gently stir at room temperature in the sealed plastic bottle overnight. The solution was drop-cast onto a clean, dry quartz microscope slide and was allowed to dry overnight in a UV-proof room. The “Before UV” samples were analyzed upon drying, and the “After UV” samples were polymerized in a 254 nm UV crosslinker chamber (power = 7.6 mW cm^−2^) for ten minutes prior to analysis.

#### 3.1.2. Stimuli Treatment of Films

Films that underwent stimuli treatment are denoted by “UV mx Heat mx”, where “UV” = 254 nm exposure for 10 min at room temperature, “Heat” = 150 °C for 1 h, and m = # of treatments by “UV” and/or “Heat”.

Thermal treatment: Films that underwent thermal treatment were heated to 150 °C for 1 h in air.

UV polymerization: Solid-state polymerization by UV irradiation of DCDDA and the 2:1 PDI-mono(*n*-imz)/DCDDA drop-cast films was performed in a Fisher Scientific UV Crosslinker equipped with 254 nm bulbs at a power of 7.6 mW cm^−2^ for 10 min at room temperature. 

### 3.2. Instrumentation

**Nuclear magnetic resonance** (**NMR**) **spectroscopy:** ^1^H NMR spectra were recorded on a Bruker DMX 500 MHz NMR spectrometer at 24° C. Deuterated chloroform (CDCl_3_) was used as a solvent with the reference peak at 7.24 ppm. Deuterated tetrahydrofuran (THF-d_8_) was used as a solvent with the reference peak at 3.58 ppm.

**Infrared** (**IR**) **spectroscopy:** Infrared spectra were acquired on a Nicolet Magna-IR 560 spectrometer with a resolution of 2 cm^−1^ and 32 scans in micro-ATR mode. The spectrometer was equipped with a Specac Quest single-reflection ATR accessory containing a diamond crystal sample plate.

**Dynamic scanning calorimetry** (**DSC**)**:** The indium standard-calibrated TA-2920 DSC (Q-100 series) instrument was used to analyze the thermal properties of DCDDA, PDI-mono(*n*-imz) small molecules, and the 2:1 PDI-mono(*n*-imz)/DCDDA drop-cast films before and after UV irradiation. The amount of sample was 5–10 mg, with a scanning rate of 5 °C/min. Phase transition temperatures were determined using TA Universal Analysis software v5.5.24. The first heat cycle was used for the PDI-mono(*n*-imz) small molecules, and the second heat cycle was used in determining the phase transition temperature of DCDDA after UV irradiation, as well as for the **2:1 PDI-mono**(***n*-imz**)**/DCDDA** drop-cast films before and after UV irradiation.

**Small-angle X-ray scattering** (**SAXS**)**:** SAXS was performed on a pin-hole collimated Bruker Nano STAR instrument configured with Cu-Kα radiation (1.5418 Å). The scattered intensity was recorded on a Mikro Gap VÅNTEC-2000 detector (pixel size = 67 mm) with a sample-to-detector distance of ~67 cm to cover a scattering vector, $q\equiv \frac{4\pi }{\lambda }\sin\frac{\theta }{2}$, ranging from 0.007 to 0.015 to 0.462 Å^−1^. Silver behenate was used to calibrate the sample-to-detector distance. The samples were prepared by sandwiching them between two pieces of scotch tape. A scotch tape substrate blank was analyzed to subtract the background from the SAXS patterns.

**Wide-angle X-ray scattering** (**WAXS**)**:** Oxford Diffraction XCalibur PX Ultra (transmission mode) was used for the WAXS measurements with an Onyx detector (CuKα radiation 1.5418 Å, double mirror focusing, 35 kV and 35 mA). The samples were prepared by sandwiching them between two pieces of scotch tape. A scotch tape substrate blank was analyzed to subtract the background from the WAXS patterns.

**UV–Vis absorbance:** UV–Vis analysis of the drop-cast films of DCDDA after UV irradiation, PDI-mono(*n*-imz) small molecules, and drop-cast films of **2:1 PDI-mono**(***n*-imz**)**/DCDDA** before and after UV irradiation was performed on a Shimadzu UV-Vis Spectrometer (UV-2450) in absorbance mode with a wavelength range of 300–800 nm.

**Electrical conductivity measurement by four-point probe:** The Ossila four-point probe system (UK, T2001A, software version 1.0) was used to measure the sheet resistance, resistivity, and conductivity of the films. To reduce the impact of humidity on the ionic conductivity, we performed our experiments in a lab with a dehumidifier that served to reduce the relative humidity present in the room. Additionally, the experiments were performed at room temperature with a marginal error of (±2 °C).

The ionic conductivity of the **x:y PDI-mono**(**n-imz**)**/DCDDA** lamellar structures was assessed with the probes vertically hooked onto a horizontally lying drop-cast film. Several measurements from different regions of the film were recorded, and the average values were reported.

The 4-point probe has a space distance of 1.27 mm between each probe. Initially, the 4-point probe instrument was calibrated by using an ITO glass (2 × 1.5 cm, 100 nm thickness) substrate, which had a resistance of 18 Ω/sq, resistivity of 1.8 µΩ/sq, and conductivity of 554.6 kS/m. The I–V curve and data were directly obtained from the instrument, and then the sheet resistance (R), resistivity, and conductivity values were obtained from the Ossila software version 1.0 by applying the sample and probe parameters. The thickness of ionic gels was measured using a micrometer vernier caliper. The resistance, resistivity, and conductivity of the sheet were calculated using the following formulas:

Rs = 4.5323 ∆V/I ρ = Rs × t σ = 1/ρ

where ΔV is the change in voltage measured between the inner probes, I is the current applied between the outer probes, Rs is the resistance of the sheet (Ω/sq), ρ is the resistivity (Ω m), σ is the conductivity (S/m), and t is the film thickness (µm) measured using a micrometer vernier caliper. The thickness of each gel varied from one sample to another. To obtain accurate data, the measurements were taken from the center of the sample or sheet in triplicates.

## 4. Conclusions

A novel approach to supramolecular PDI-PDA polymeric systems has been developed from a DA diacid monomer hydrogen-bonded to imidazole-appended PDI molecules that are π-π-stacked. Here, we elucidated the importance of the composition of the imidazole-appended perylene diimide of varying methylene spacer length (*n* = 3, 4, and 6) and 10, 12 docosadiynedioic acid in the ratio of 2:1 to the supramolecular self-assembly, final morphology, and properties. The supramolecular formation was monitored in solution by ^1^H NMR spectroscopy and revealed a deviation from the theoretical 2:1 **PDI-mono**(***n*-imz**)**/DCDDA** to an experimental 2:1.25 molar ratio. FTIR spectroscopy of the supramolecular structures in the solid state displayed retention of the supramolecular formation when comparing the characteristic C=O stretching in the pure **DCDDA** monomer to the supramolecular structures. UV–Vis spectroscopy was used to study various chromatic transitions, where topochemical polymerization of the drop-cast films proceeded upon UV radiation for 10 min, resulting in blue-phase PDCDDA formation. Subsequent thermal treatment displayed thermochromic behavior upon the blue→red phase formation in the films. Interestingly, thermal polymerization of the blue or red phase did not occur. Thermal studies by DSC showed a dual dependence of the methylene spacer length and stimuli treatment (UV or heat) on characteristic transitions in the films. Furthermore, small-angle X-ray scattering (SAXS) and wide-angle X-ray scattering (WAXS) showed well-defined hierarchical semiconducting nanostructures with interconnected “chessboard”-patterned lamellar stacking. This unusual morphology with domain connectivity directly enhanced the ionic conductivity of this ionic liquid-doped 2:1 system compared to our previously published 1:1 composition of PDI and PDCDDA. Thus, the rational design of this 2:1 system with a tailored composition, architecture, and self-assembly will be important for energy applications involving the transport of ions and charges.

## Figures and Tables

**Table 1:** SAXS peaks (Figure 6), q_n_*^,^ and the corresponding d-spacing of the 2:1 **PDI-mono***(***4*-*imz**)**/PDCDDA** drop-cast films.

Peak Assignment	q (Å^−1^)	d-Spacing (Å) from the 1st Order
q_1_*: the 1st-order Bragg reflection along the *a* axis (indicating the molecular length)	0.149	42.1
q_2_*: the 1st-order Bragg reflection interlamellar distance	0.178	35.3
q_3_*: the 1st-order Bragg reflection indicating the repeat spacing along the *b* axis.	0.210	29.9
q_4_*: the 1st-order Bragg reflection indicating the repeat spacing along the *l* axis	0.240	26.2
q_5_*: Bragg reflection of the (1,1) plane	0.253	-
q_6_*: the 2nd-order reflection for q_1_^*^	0.293	-
q_7_*: the 2nd-order reflections for q_2_^*^	0.353	-
q_8_*: from PDI-mono(4-imz) polymer	0.384	-

**Table 2:** Ionic conductivity values of **1:1 PDI-mono**(***4*-imz**)**/PCDDA** and **2:1 PDI-mono**(**4-imz**)**/PDCDDA** before **UV** exposure in the presence of ionic liquid.

Sample Name	Applied Outer Current (mA)	Measured Voltage (mV)	Sheet Resistance (Ohms/Square)	Resistivity (Ohm.cm)	Conductivity (mS/cm)
**1:1 PDI-mono**(***4*-imz**)**/PCDDA**	3.20 × 10^−3^	3.78	4077.862	4.0777	2.610 × 10^−3^
**2:1 PDI-mono**(***4*-imz**)**/PCDDA**	3.6 × 10^−3^	2.01	2100.333	2.1003	4.994 × 10^−3^

## Data Availability

Data are contained within the article and Supplementary Materials.

## Supplementary Materials

The following supporting information can be downloaded at: https://www.mdpi.com/article/10.3390/molecules30061207/s1, Figures S1 and S2: NMR spectra of various **2:1 PDI-mono(*n*-imz)/DCDDA**, Figures S3 and S4: FTIR-ATR spectra of various **2:1 PDI-mono(*n*-imz)/DCDDA**, Figures S5–S7: UV-visible spectra of **2:1 PDI-mono(*n*-imz)/DCDDA** before and after UV or thermal treatment, Figure S6 and Table S1: TGA plot and numerical data for **DCDDA**, and **2:1 PDI-mono(*n*-imz)/DCDDA**, Figures S7–S12 and Table S2: DSC data and corresponding numerical data table for **DCDDA** and **2:1 PDI-mono(*n*-imz)/DCDDA** before and after UV and/or thermal treatment, Figures S13–S18 and Tables S3–S6: 1D and 2D WAXS data and corresponding peak assignments in the tables for **DCDDA** and **2:1 PDI-mono(*n*-imz)/DCDDA** before and after UV and/or thermal treatment, Figures S19–S22 and Tables S7–S10: 1D SAXS data and corresponding peaks assignemnts in tables for **DCDDA** and **2:1 PDI-mono(*n*-imz)/DCDDA** before and after UV and/or thermal treatment, Table S11 and Table S12: Ionic conductivity for **1:1 PDI-mono(*4*-imz)/PCDDA** and **2:1 PDI-mono(*4*-imz)/PDCDDA** in ionic liquids after UV exposure and various control samples.
