# Supplementary material for: Nanoscale “Chessboard” Pattern Lamellae in a Supramolecular Perylene-Diimide Polydiacetylene System
Source: Molecules. 2025 Mar 7;30(6):1207. doi: 10.3390/molecules30061207 (PMC11946615; doi:10.3390/molecules30061207)
Supplement: Supplementary file 1 [file molecules-30-01207-s001.zip › molecules-3483930-supplementary.pdf]

Electronic Supporting Information

Nanoscale “Chessboard” Pattern Lamellae in a Supramolecular Perylene-Diimide Polydiacetylene System

Ian J. Martin 1,†, Francis Kiranka Masese 1,†, Kuo-Chih Shih 2,3, Mu-Ping Nieh 2,3,4,5,* and Rajeswari M. Kasi 1,2,3,*

1 Department of Chemistry, University of Connecticut, Storrs, CT 06269, USA
2 Polymer Program, University of Connecticut, Storrs, CT 06269, USA
3 Institute of Materials Science, University of Connecticut, Storrs, CT 06269, USA
4 Department of Chemical and Biomolecular Engineering, University of Connecticut, Storrs, CT 06269, USA
5 Biomedical Engineering, University of Connecticut, Storrs, CT 06269, USA
* Correspondence: mu-ping.nieh@uconn.edu (M.-P.N.); rajeswari.kasi@uconn.edu (R.M.K.); Tel.: +1-(860)-486-4713 (R.M.K.)
† These authors contributed equally to this work.

Table of Contents

Section	*Page*
^{1}H NMR overlays	S2-3
FTIR-ATR spectra	S4-5
UV-Vis absorbance spectra	S6-7
TGA thermograms	S8
DSC data	S9-13
1D and 2D WAXS data	S14-21
1D SAXS data	S22-29
Ionic Conductivity data	S30

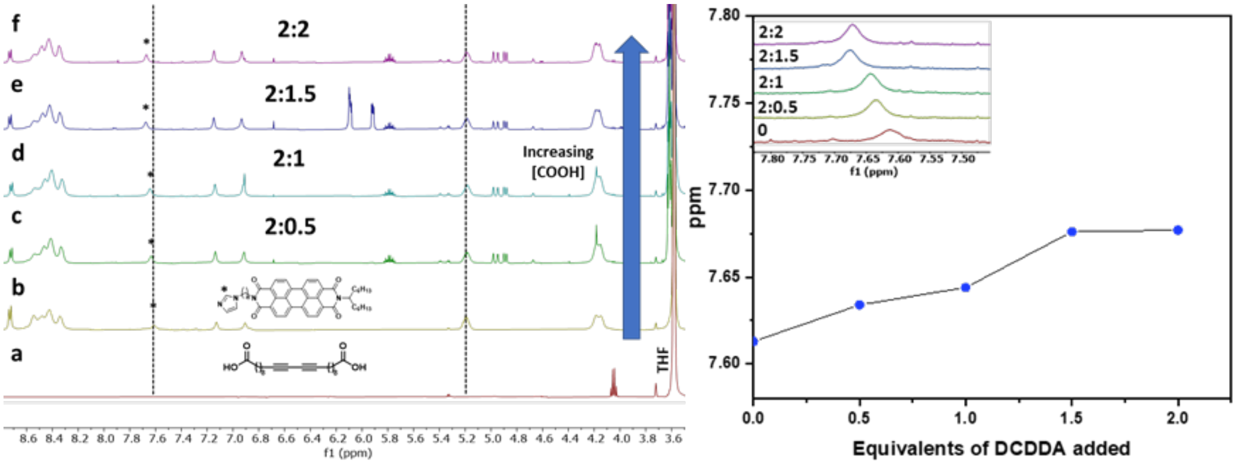

Figure S1. (Left) ^{1}H NMR spectra overlay of (a) **DCDDA** (b) **PDI-mono(3-imz)** (c) 2:0.5 **PDI-mono(3-imz)/DCDDA** (d) 2:1 **PDI-mono(3-imz)/DCDDA** (e) 2:1.5 **PDI-mono(3-imz)/DCDDA** (f) 2:2 **PDI-mono(3-imz)/DCDDA** in THF-d_8 at 25°C. The imidazole proton that is denoted by an asterisk (*) shifts from 7.61 ppm to 7.68 ppm and the tertiary proton chemical shift at 5.18 ppm does not shift upon hydrogen bonding. (Right) Chemical shift change of PDI aromatic imidazole proton (labeled with * in ^{1}H NMR spectra overlay) upon addition of increasing amount of **DCDDA**. Points represent experimental data. Inset shows ^{1}H NMR spectra overlay of aromatic imidazole proton peak from 7.82 ppm to 7.48 ppm of **PDI-mono(3-imz)/DCDDA** with increasing amounts of **DCDDA**.

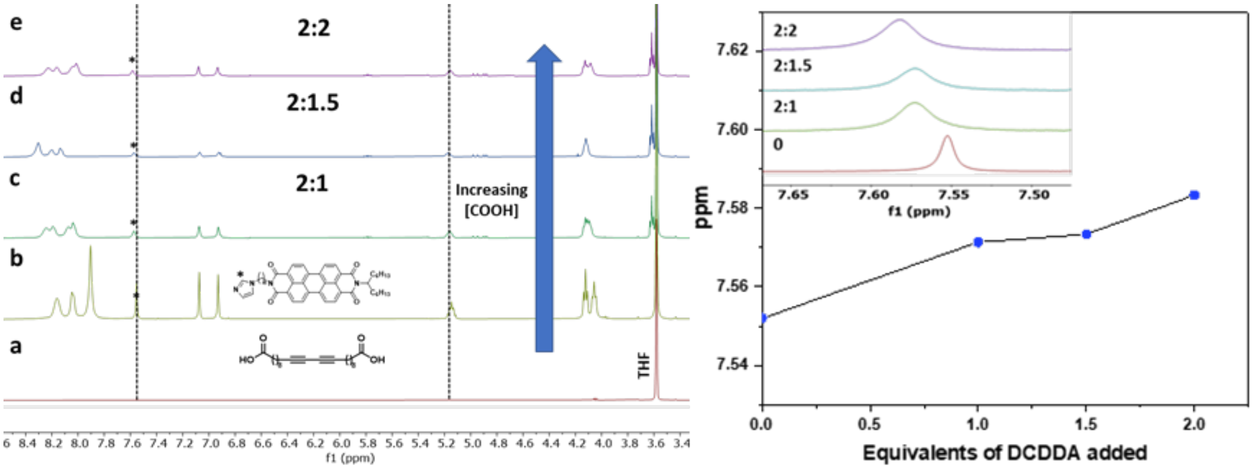

Figure S2. (Left) ^{1}H NMR spectra overlay of (a) **DCDDA** (b) **PDI-mono(4-imz)** (c) 2:1 **PDI-mono(4-imz)/DCDDA** (d) 2:1.5 **PDI-mono(4-imz)/DCDDA** (e) 2:2 **PDI-mono(4-imz)/DCDDA** in THF-d_8 at 25°C. The imidazole proton that is denoted by an asterisk (*) shifts from 7.55 ppm to 7.58 ppm and the tertiary proton chemical shift at 5.18 ppm does not shift upon hydrogen bonding. (Right) Chemical shift change of PDI aromatic imidazole proton (labeled with * in ^{1}H NMR spectra overlay) upon addition of increasing amount of **DCDDA**. Points represent experimental data. Inset shows ^{1}H NMR spectra overlay of aromatic imidazole proton peak from 7.66 ppm to 7.48 ppm of **PDI-mono(4-imz)/DCDDA** with increasing amounts of **DCDDA**.

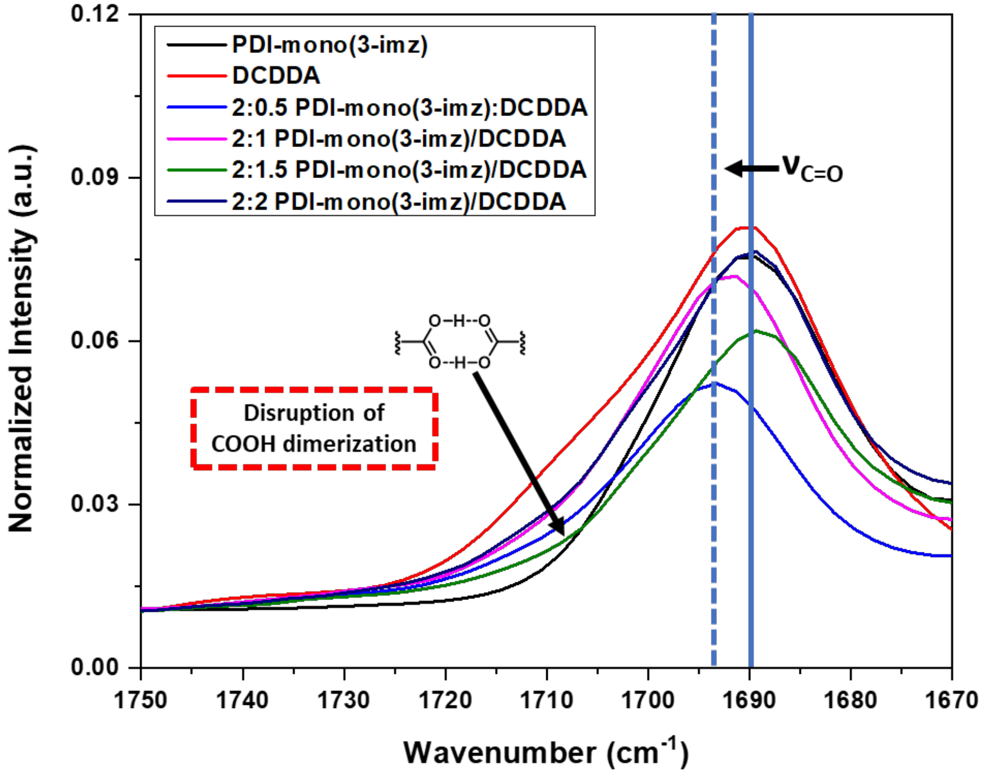

Figure S3. FTIR-ATR spectra overlay of **PDI-mono(3-imz)** (black line) **DCDDA** (red line) 2:0.5 **PDI-mono(3-imz)**/**DCDDA** (blue line) 2:1 **PDI-mono(3-imz)**/**DCDDA** (pink line) 2:1.5 **PDI-mono(3-imz)**/**DCDDA** (green line) 2:2 **PDI-mono(3-imz)**/**DCDDA** (purple line) powder at 25°C.

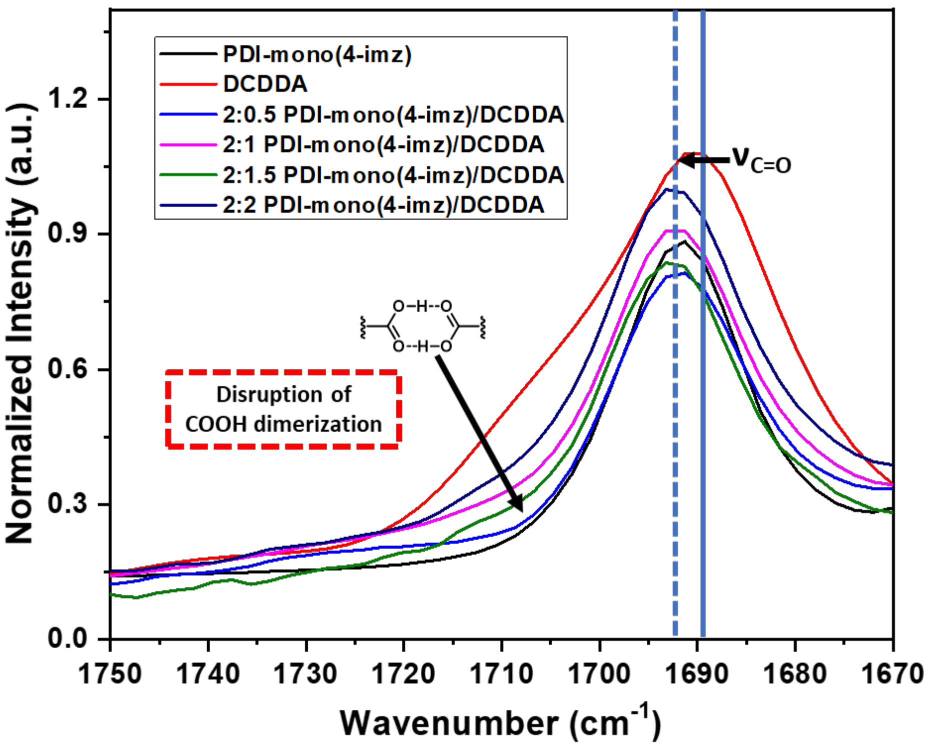

Figure S4. FTIR-ATR spectra overlay of **PDI-mono(4-imz)** (black line) **DCDDA** (red line) 2:0.5 **PDI-mono(4-imz)**/**DCDDA** (blue line) 2:1 **PDI-mono(4-imz)**/**DCDDA** (pink line) 2:1.5 **PDI-mono(4-imz)**/**DCDDA** (green line) 2:2 **PDI-mono(4-imz)**/**DCDDA** (purple line) powder at 25°C.

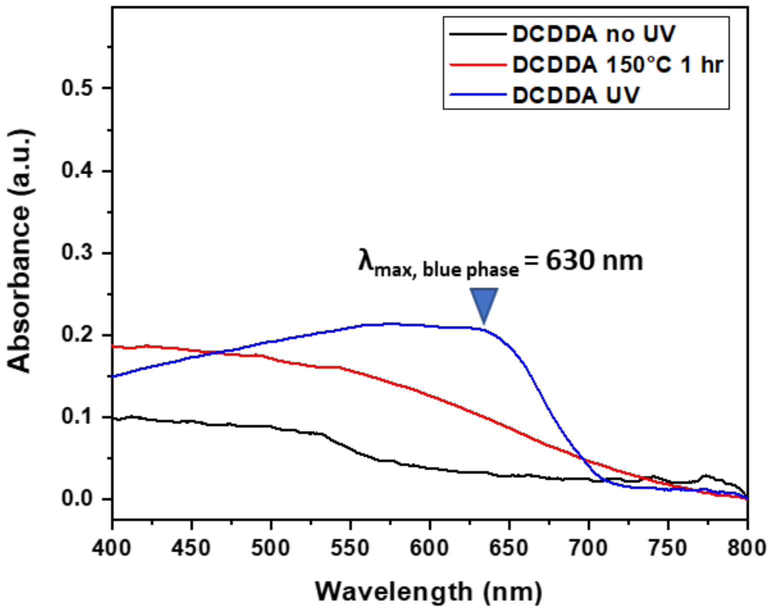

Figure S5. UV-Vis spectra overlay of **DCDDA** powder before UV (black line), heat treatment (red line), UV radiation (blue line).

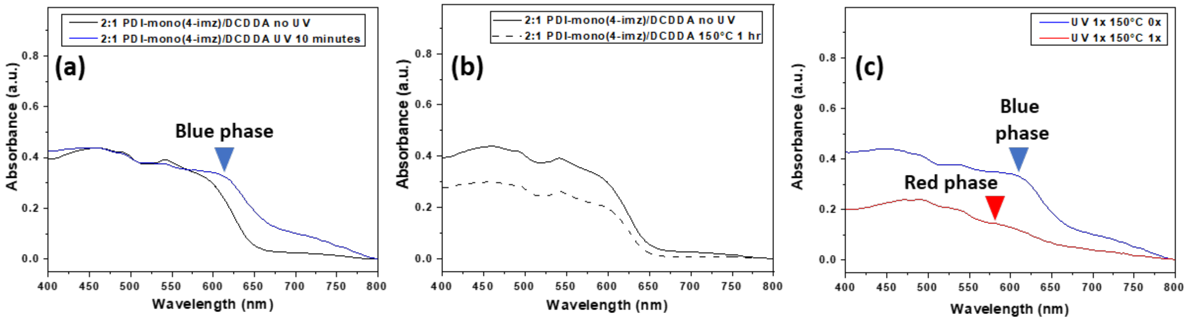

Figure S6. UV-Vis absorbance spectra overlays of 2:1 **PDI-mono(4-imz)/DCDDA** drop-cast films indicating **(a)** blue phase PDA formation after UV irradiation **(b)** no PDA formation after thermal treatment to 150 °C for 1 hr **(c)** blue to red phase PDA transformation upon after thermal treatment to 150 °C for 1 hr.

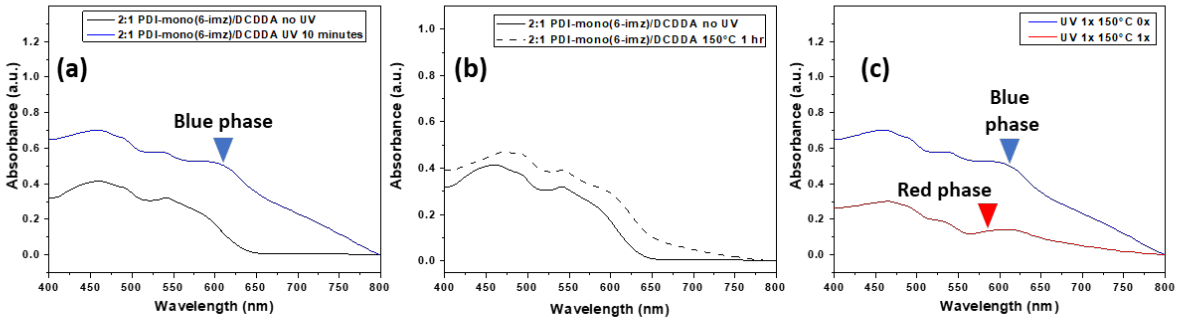

Figure S7. UV-Vis absorbance spectra overlays of 2:1 **PDI-mono(6-imz)/DCDDA** drop-cast films indicating **(a)** blue phase PDA formation after UV irradiation **(b)** no PDA formation after thermal treatment to 150 °C for 1 hr **(c)** blue to red phase PDA transformation upon after thermal treatment to 150 °C for 1 hr.

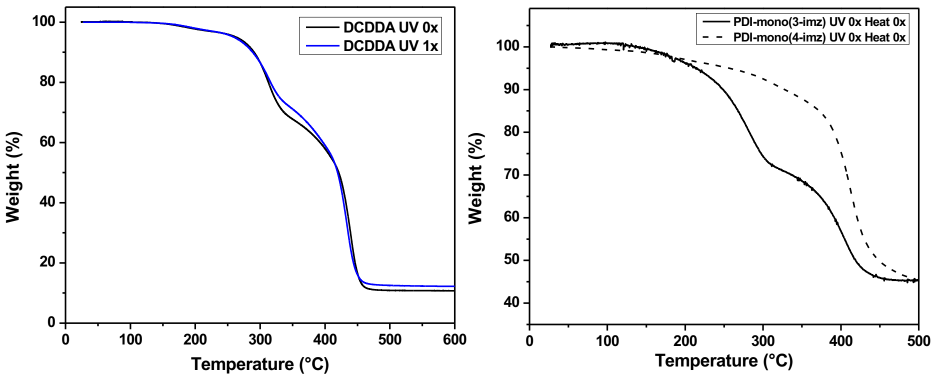

Figure S8. TGA thermogram overlays of (Left) **DCDDA** before (black line, UV 0x) and after UV (blue line, UV 1x). (Right) 2:1 **PDI-mono(*n*-imz)/DCDDA** (*n* = 3, 4) drop-cast films before exposure to various stimuli. Decomposition temperatures, T_d, can be found in **Table S1**.

	**Decomposition temperature, T_d (°C)[a]**	
	**UV 0x Heat 0x**	**UV 1x Heat 0x**
**DCDDA**	249.4	247.9
**2:1 PDI-mono(3-imz)/DCDDA**	207.4	-
**2:1 PDI-mono(4-imz)/DCDDA**	229.8	-

Table S1. Data containing decomposition temperatures, T_d, measured at 96 wt %[a] of **DCDDA**, and 2:1 **PDI-mono*(n*-imz)/DCDDA** where *n* = 3 or 4 as a function of stimuli treatment.

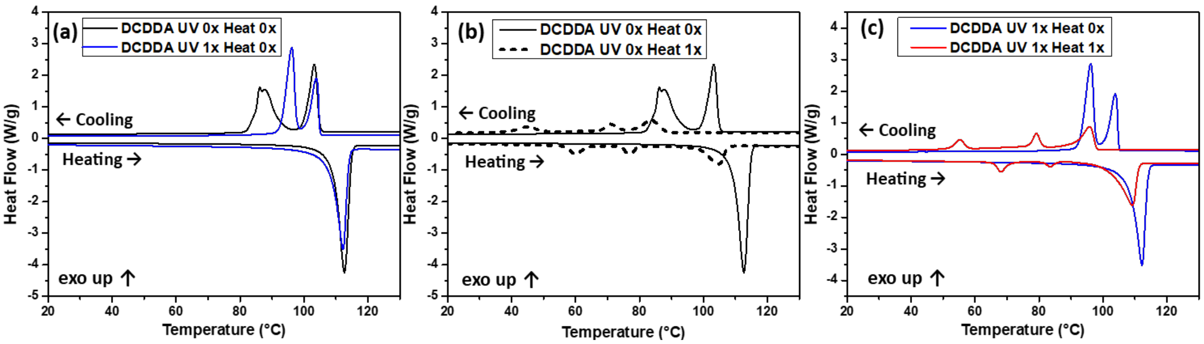

Figure S9. DSC thermograms of **DCDDA** powder before and after exposure to various stimuli. **(a)** Before and after UV (black line and blue line, respectively) **(b)** Before and after heating (black line and dashed black line, respectively) and **(c)** After UV and after UV and heating (blue line and red line, respectively).

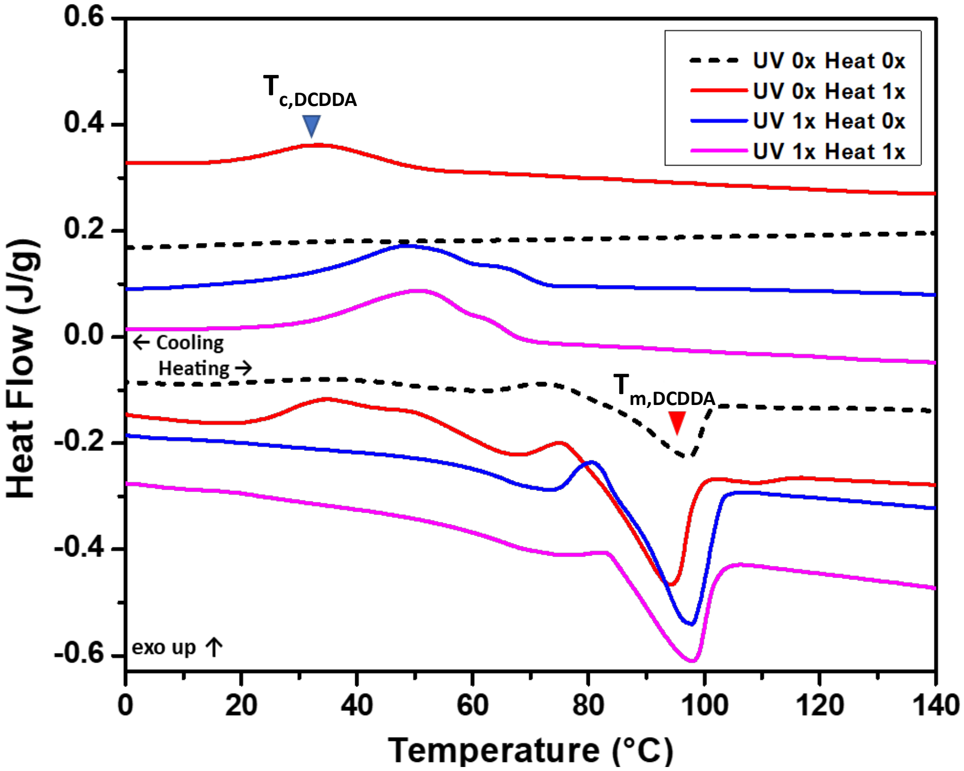

Figure S10. DSC thermogram overlay of 2:1 **PDI-mono(3-imz)/DCDDA** drop-cast films before and after exposure to various stimuli. Melting transition temperature ($T_{m,DCDDA}$) is labeled with a red triangle and the crystallization transition temperature ($T_{c,DCDDA}$) is labeled with a blue triangle; only one of the samples is labeled with $T_{c,DCDDA}$ or $T_{m,DCDDA}$ for clarity.

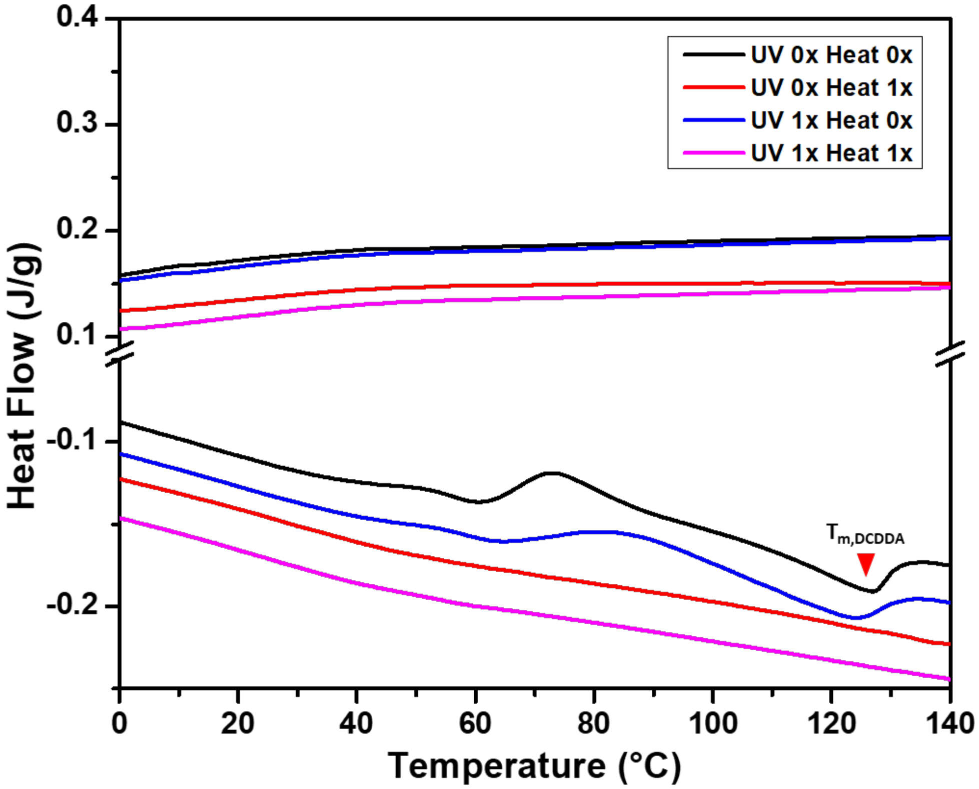

Figure S11. DSC thermogram overlay of 2:1 **PDI-mono(4-imz)/DCDDA** drop-cast films before and after exposure to various stimuli. Melting transition temperature ($T_{m,DCDDA}$) is labeled with a red triangle; only one of the samples is labeled with $T_{m,DCDDA}$ for clarity.

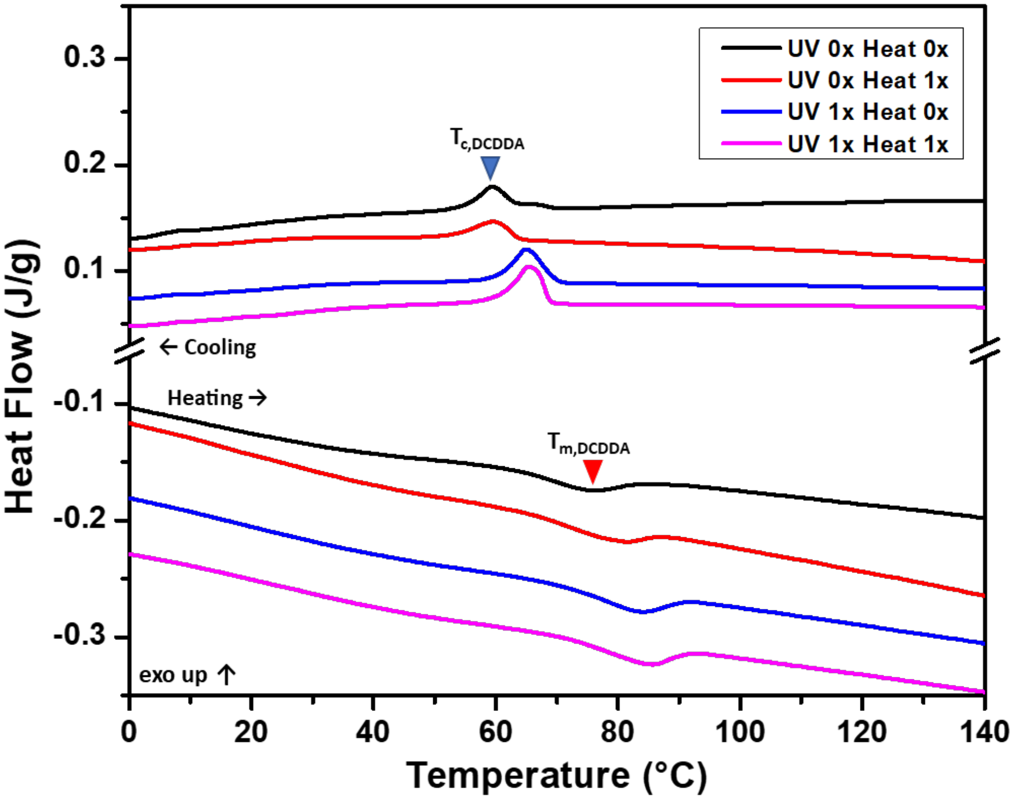

Figure S12. DSC thermogram overlay of 2:1 **PDI-mono(6-imz)/DCDDA** drop-cast films before and after exposure to various stimuli. Melting transition temperature ($T_{m,DCDDA}$) is labeled with a red triangle and the crystallization transition temperature ($T_{c,DCDDA}$) is labeled with a blue triangle; only one of the samples is labeled with $T_{c,DCDDA}$ or $T_{m,DCDDA}$ for clarity.

$T_{m,DCDDA}$ (°C), $\Delta H_{m,DCDDA}$ (J/g) / ($T_{c,DCDDA}$ (°C), $\Delta H_{c,DCDDA}$ (J/g))				
n	**UV 0x Heat 0x**	**UV 0x Heat 1x**	**UV 1x Heat 0x**	**UV 1x Heat 1x**
**3**	97.1, 15.3 / (NA, NA)	94.0, 30.6 / (34.0, 8.9)	97.7, 36.3 / (48.5, 25.1)	97.9, 24.5 / (50.9, 24.8)
**4**	126.6, 3.3 / (NA, NA)	NA, NA / (NA, NA)	122.5, 5.7 / (NA, NA)	NA, NA / (NA, NA)
**6**	74.5, 1.6 / (59.4, 1.9)	78.6, 1.3 / (59.6, 1.3)	83.2, 1.9 / (65.0, 2.3)	84.9, 2.0 / (65.4, 2.4)

Table S2. Melting transition temperatures ($T_{m,DCDDA}$), enthalpies of melting transition ($\Delta H_{m,DCDDA}$), crystallization transition temperatures ($T_{c,DCDDA}$), and enthalpies of crystallization transition ($\Delta H_{c,DCDDA}$) as a function of methylene spacer length, *n*, and stimuli treatment of **DCDDA** regions in 2:1 **PDI-mono(*n*-imz):DCDDA** drop-cast films. Data derived from DSC thermograms in **Figures S10-12** and from the second heat cycle at a rate of 5°C min^{-1}.

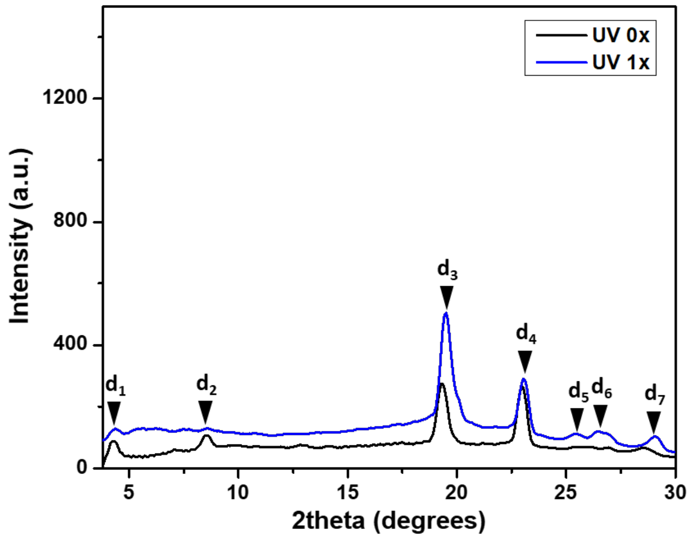

Figure S13. 1D WAXS spectra overlay of **DCDDA** before and after exposure to UV ("UV" = 254 nm exposure for 10 minutes at room temperature). Before UV (black line), after UV (blue line). WAXS peak assignments can be found in **Table S3**.

	**DCDDA d-spacing (d_n)**	
**Peak Assignment**	**UV 0x**	**UV 1x**
**DCDDA (200), d_1 (Å)**	20.65	20.25
**DCDDA (400), d_2 (Å)**	10.36	10.31
**DCDDA backbone periodicity, d_3 (Å)**	4.59	4.55
**DCDDA π-π stacking, d_4 (Å)**	3.86	3.85
**DCDDA, d_5 (Å)**	NA	3.50
**DCDDA (600), d_6 (Å)**	3.31	3.37
**DCDDA, d_7 (Å)**	3.12	3.07

Table S3. Numerical values for WAXS peaks, d_n, and their corresponding d-values (Å), found in **Figure S13** of **DCDDA** powder before and after exposure to 254 nm UV radiation for 10 minutes at room temperature.

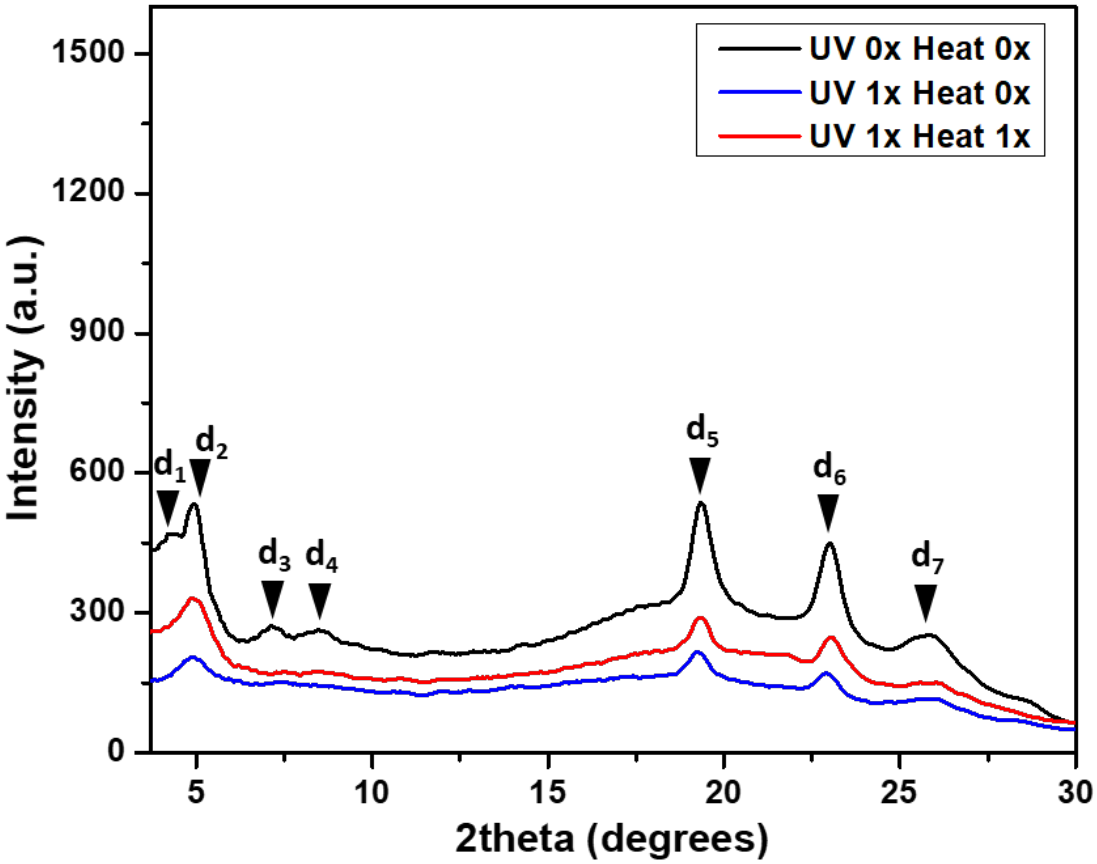

Figure S14. 1D WAXS spectra overlay of 2:1 **PDI-mono(3-imz)/DCDDA** before and after exposure to various stimuli. Before UV (black line), after UV (blue line), and after UV and heating (red line). WAXS peak assignments can be found in **Table S4**.

	2:1 PDI-mono(3-imz)/DCDDA d-spacing (d_n)		
**Peak Assignment**	**UV 0x Heat 0x**	**UV 1x Heat 0x**	**UV 1x Heat 1x**
**DCDDA (200), d_1 (Å)**	20.65	NA	NA
**PDI/DCDDA cylinder (002), d_2 (Å)**	17.93	17.93	17.93
**PDI/DCDDA cylinder (003), d_3 (Å)**	12.37	11.94	11.73
**DCDDA (400), d_4 (Å)**	10.42	10.53	10.57
**DCDDA backbone periodicity, d_5 (Å)**	4.58	4.61	4.59
**DCDDA π-π stacking, d_6 (Å)**	3.86	3.88	3.85
**PDI π-π stacking, d_7 (Å)**	3.45	3.45	3.49

Table S4. Numerical values for WAXS peaks, d_n, and their corresponding d-values (Å), found in **Figure S14** of 2:1 **PDI-mono(3-imz)/DCDDA** drop-cast films before and after exposure to various stimuli.

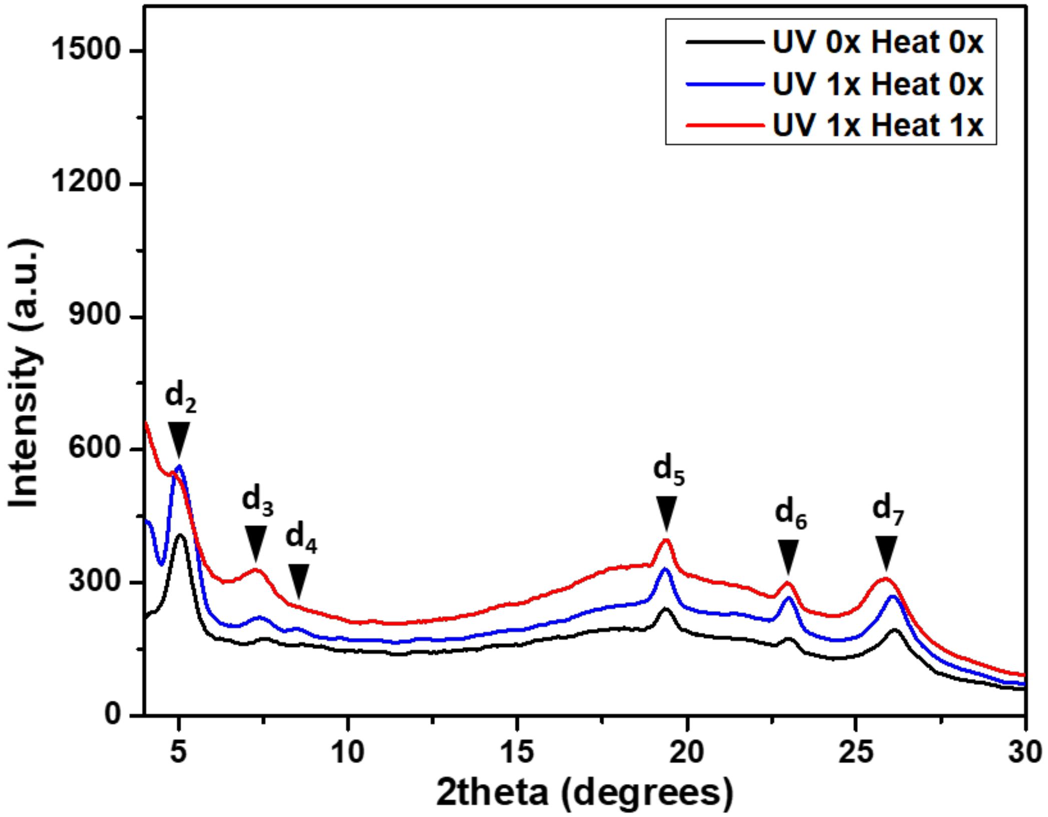

Figure S15. 1D WAXS spectra overlay of 2:1 **PDI-mono(4-imz)/DCDDA** before and after exposure to various stimuli. WAXS peak assignments can be found in **Table S5**.

	2:1 PDI-mono(4-imz)/DCDDA d-spacing (d_n)		
**Peak Assignment**	**UV 0x Heat 0x**	**UV 1x Heat 0x**	**UV 1x Heat 1x**
**PDI/DCDDA cylinder (002), d_2 (Å)**	17.46	17.61	18.24
**PDI/DCDDA cylinder (003), d_3 (Å)**	11.67	12.01	12.08
**DCDDA (400), d_4 (Å)**	10.20	10.36	NA
**DCDDA backbone periodicity, (d_5 (Å)**	4.58	4.58	4.58
**DCDDA π-π stacking, d_6 (Å)**	3.86	3.86	3.87
**PDI π-π stacking, d_7 (Å)**	3.41	3.41	3.44

Table S5. Numerical values for WAXS peaks, d_n, and their corresponding d-values (Å), found in **Figure S15** of 2:1 **PDI-mono(4-imz)/DCDDA** drop-cast films before and after exposure to various stimuli.

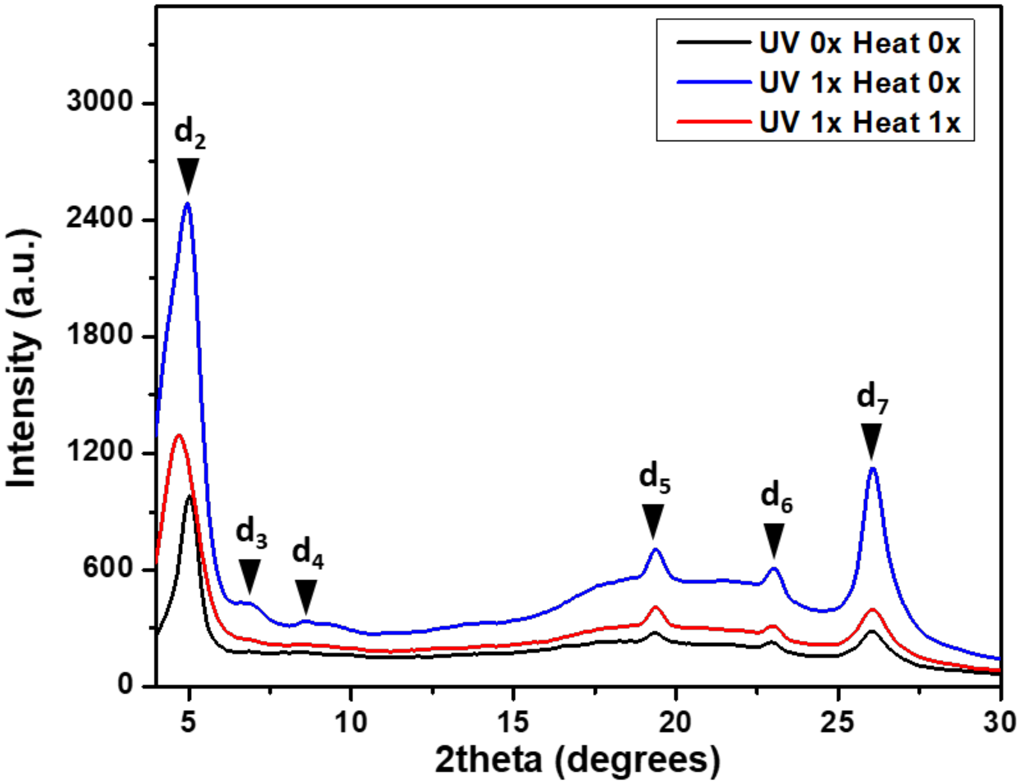

Figure S16. 1D WAXS spectra overlay of 2:1 **PDI-mono(6-imz)/DCDDA** before and after exposure to various stimuli. WAXS peak assignments can be found in **Table S6**.

	2:1 PDI-mono(6-imz)/DCDDA d-spacing (d_n)		
**Peak Assignment**	**UV 0x Heat 0x**	**UV 1x Heat 0x**	**UV 1x Heat 1x**
**PDI/DCDDA cylinder (002), d_2 (Å)**	17.61	17.93	18.74
**DCDDA backbone periodicity, (d_5 (Å)**	4.58	4.58	4.58
**DCDDA π-π stacking, d_6 (Å)**	3.87	3.85	3.87
**PDI π-π stacking, d_7 (Å)**	3.42	3.41	3.42

Table S6. Numerical values for WAXS peaks, d_n, and their corresponding d-values (Å), found in **Figure S16** of 2:1 **PDI-mono(6-imz)/DCDDA** drop-cast films before and after exposure to various stimuli.

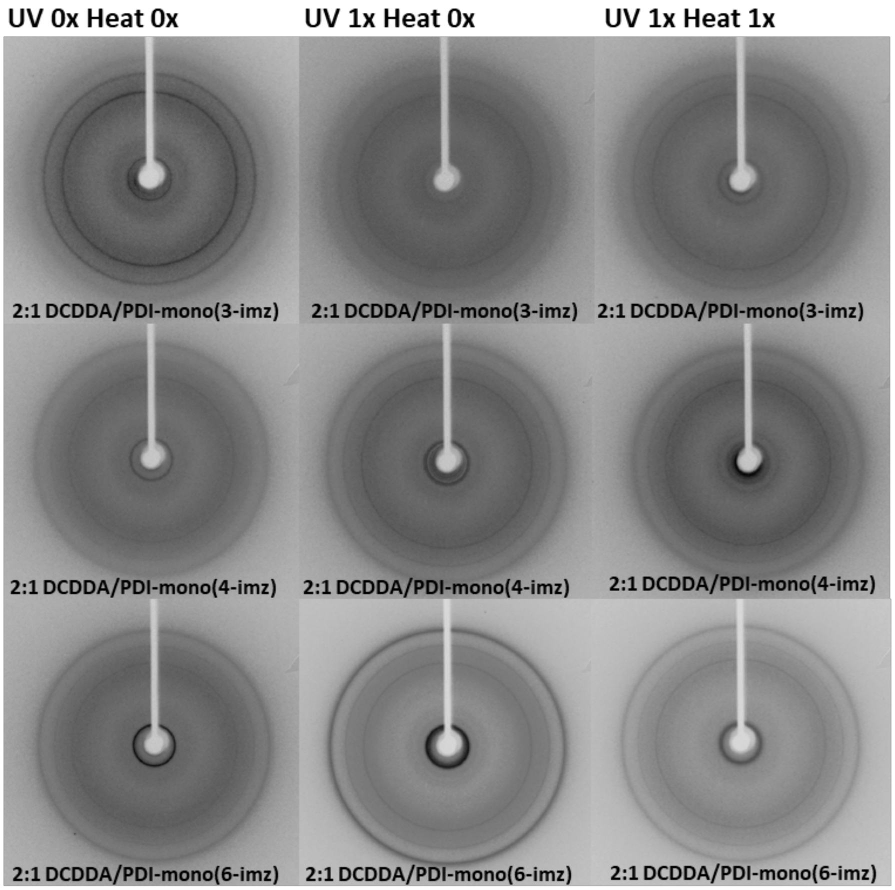

Figure S17. 2D WAXS images of 2:1 **PDI-mono(*n*-imz)/DCDDA**, where *n* = 3,4, or 6, before and after exposure to various stimuli. Corresponding 1D WAXS spectra can be found in **Figures S14-16** and numerical data in **Tables S4-6**.

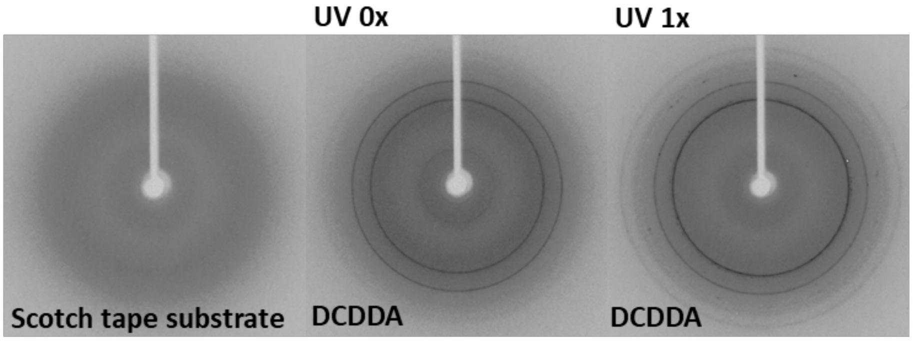

Figure S18. 2D WAXS images of the scotch tape substrate used in WAXS and SAXS experiments showing no distinct crystalline peaks (left) and **DCDDA** before (middle) and after (right) UV treatment. Corresponding 1D WAXS spectra to **DCDDA** can be found in **Figure S13** and numerical data in **Tables S3**.

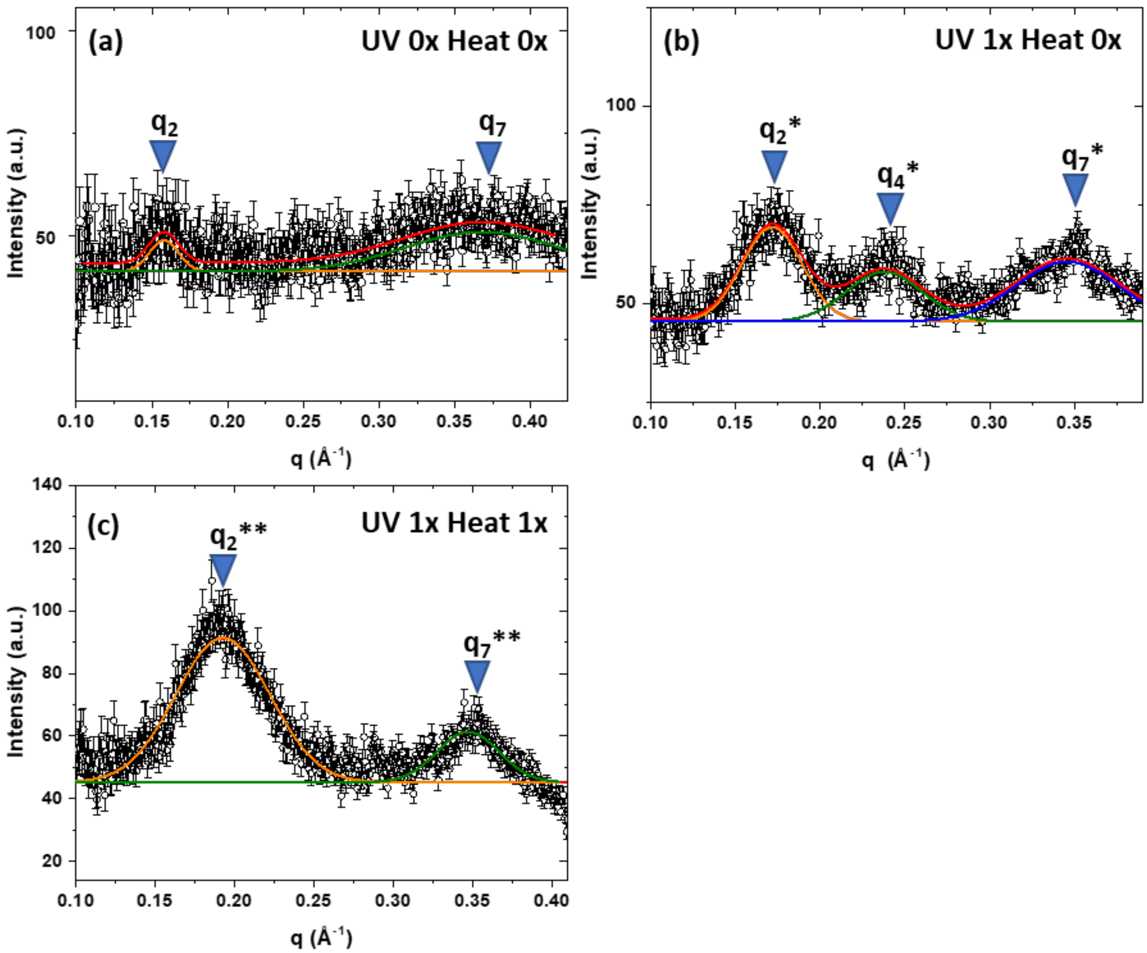

Figure S19. 1D SAXS spectra of 2:1 **PDI-mono(3-imz)/DCDDA** after various stimuli: **(a)** UV 0x Heat 0x **(b)** UV 1x Heat 0x **(c)** UV 1x Heat 1x. SAXS peak assignments (q_n) can be found in **Table S7**. Peak positions were determined using the fitting function in Origin.

	2:1 PDI-mono(3-imz)/DCDDA		
**Peak Assignment, n**	$\mathbf{q_n}$ (Å^{-1}), ($\mathbf{d_n}$ (Å))	$\mathbf{q_n}^*$ (Å^{-1}), ($\mathbf{d_n}^*$ (Å))	$\mathbf{q_n}^{**}$ (Å^{1}), ($\mathbf{d_n}^{**}$ (Å))
$\mathbf{q_1}$***:** the 1st order Bragg reflection along the *a*-axis (indicating the molecular length)	-	-	-
$\mathbf{q_2}$***:** the 1st order Bragg reflection interlamellar distance	0.158, (39.87)	0.172, (36.53)	0.193, (32.56)
$\mathbf{q_3}$***:** the 1st order Bragg reflection indicating the repeat spacing along *b*-axis.	-	-	-
$\mathbf{q_4}$***:** the 1st order Bragg reflection indicating the repeat spacing along the *l* axis.		0.238, (26.40)	
$\mathbf{q_5}$***:** Bragg reflection of (1,1) plane	-	-	-
$\mathbf{q_6}$***:** The 2nd order reflection for q_1*	-	-	-
$\mathbf{q_7}$***:** The 2nd order reflections for q_2*	0.368, (17.07)	0.346, (18.16)	0.347, (18.11)
$\mathbf{q_8}$***:** from PDI-mono(4-imz) Polymer	-	-	-

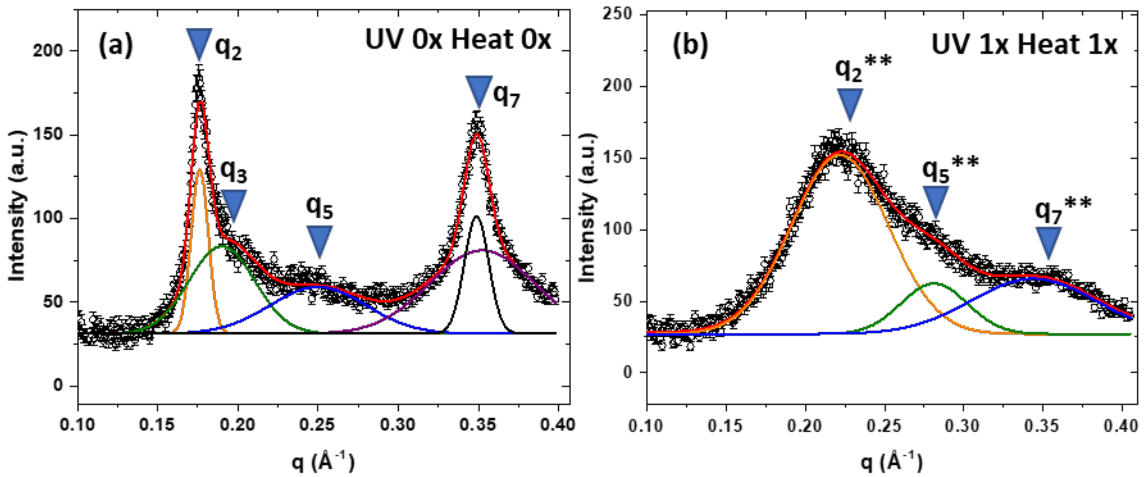

Figure S20. 1D SAXS spectra of 2:1 **PDI-mono(4-imz)/DCDDA** after various stimuli: **(a)** UV 0x Heat 0x **(b)** UV 1x Heat 1x. SAXS peak assignments (q_n) can be found in **Table S8**. Peak positions were determined using the fitting function in Origin. 1D SAXS spectrum representing "UV 1x Heat 0x" can be found in **Figure 6** of the main manuscript.

Peak Assignment, n		2:1 PDI-mono(4-imz)/DCDDA
	**q_n (Å^{-1}), (d_n (Å))**	**q_n^{**} (Å^{-1}), (d_n^{**} (Å))**
**q_1*:** the 1st order Bragg reflection along the *a*-axis (indicating the molecular length)	-	-
**q_2*:** the 1st order Bragg reflection interlamellar distance	0.176, (35.70)	0.222, (28.30)
**q_3*:** the 1st order Bragg reflection indicating the repeat spacing along *b*-axis.	0.190, (33.10)	-
**q_4*:** the 1st order Bragg reflection indicating the repeat spacing along the *l* axis.	-	-
**q_5*:** Bragg reflection of (1,1) plane	0.250, (25.13)	0.281, (22.36)
**q_6*:** The 2nd order reflection for q_1*	-	-
**q_7*:** The 2nd order reflections for q_2*	0.349, (18.00)	0.344, (18.27)
**q_8*:** from PDI-mono(4-imz) Polymer	-	-

Table S8. Numerical values for SAXS peaks q_n, q_n** (Å^{-1}), and their corresponding d-values, d_n, d_n** (Å) as a function of peak assignment, n. found in **Figure S20** of 2:1 **PDI-mono(4-imz)/DCDDA** drop-cast films. q_n/ d_n represent "UV 0x Heat 0x" and q_n**/ d_n** represent "UV 1x Heat 1x", respectively. Numerical values for SAXS peaks q_n*, d_n* representing "UV 1x Heat 0x" can be found in **Table 1** in the main manuscript.

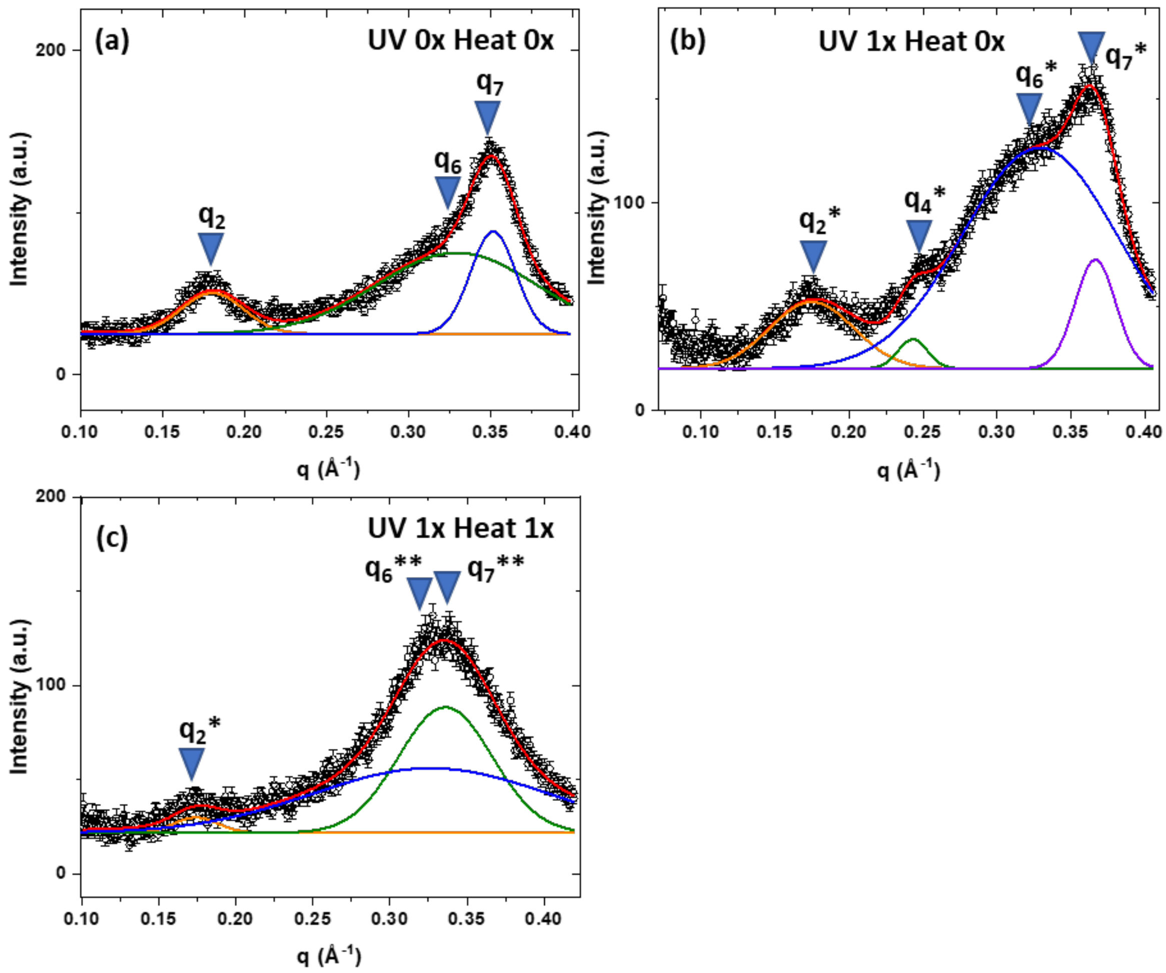

Figure S21. 1D SAXS spectra of 2:1 **PDI-mono(6-imz)/DCDDA** after various stimuli: **(a)** UV 0x Heat 0x **(b)** UV 1x Heat 0x **(c)** UV 1x Heat 1x. SAXS peak assignments (q_n) can be found in **Table S9**. Peak positions were determined using the fitting function in Origin.

	2:1 PDI-mono(6-imz)/DCDDA		
**Peak Assignment, n**	**q_n** (Å^{-1}), (**d_n** (Å))	**q_n^*** (Å^{-1}), (**d_n^*** (Å))	**q_n^{**}** (Å^{-1}), (**d_n^{**}** (Å))
**q_1*:** the 1st order Bragg reflection along the *a*-axis (indicating the molecular length)	-	-	-
**q_2*:** the 1st order Bragg reflection interlamellar distance	0.180, (34.91)	0.174, (36.11)	0.174, (36.11)
**q_3*:** the 1st order Bragg reflection indicating the repeat spacing along *b*-axis.	-	-	-
**q_4*:** the 1st order Bragg reflection indicating the repeat spacing along the *l* axis.		0.243, (25.86)	
**q_5*:** Bragg reflection of (1,1) plane	-	-	-
**q_6*:** The 2nd order reflection for q_1*	0.329, (19.10)	0.329, (19.10)	0.328, (19.18)
**q_7*:** The 2nd order reflections for q_2*	0.351, (17.07)	0.367, (17.12)	0.336, (18.70)
**q_8*:** from PDI-mono(4-imz) Polymer	-	-	-

Table S9. Numerical values for SAXS peaks q_n, q_n*, q_n** (Å^{-1}), and their corresponding d-values, d_n, d_n*, d_n** (Å) as a function of peak assignment, n. found in **Figure S21** of 2:1 **PDI-mono(6-imz)/DCDDA** drop-cast films. q_n / d_n represent "UV 0x Heat 0x", q_n*/ d_n* represent "UV 1x Heat 0x", and q_n**/ d_n** represent "UV 1x Heat 1x", respectively.

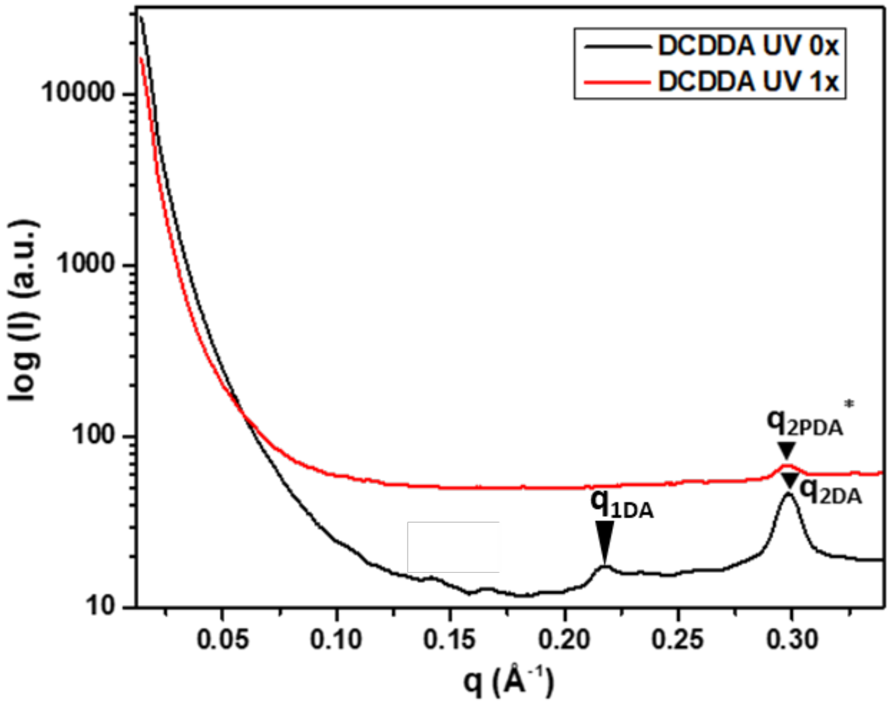

Figure S22. 1D SAXS spectra overlay of **DCDDA** powder before (black line) and after (red line) UV irradiation at 254 nm for ten minutes. SAXS peak assignments (q_{nDA} and q_{nPDA}*) can be found in **Table S10**.

	DCDDA
**q_{1DA} ($Å^{-1}$), (d_1 (Å))**	0.22, (29.02)
**q_{2DA} ($Å^{-1}$), (d_2 (Å))**	0.30, (21.07)
**q_{2PDA}^* ($Å^{-1}$), (d_2^* (Å))**	0.30, (21.09)

Table S10. Numerical values for SAXS peaks, q_{nDA}, q_{nPDA}* ($Å^{-1}$), and their corresponding d-values, d_n and d_n* (Å), found in **Figure S22** of **DCDDA** powder. q_{nDA}/ d_n represents "UV 0x" and q_{nPDA}*/ d_n* represents "UV 1x", respectively.

Sample Name	Applied outer current (mA)	Measured voltage (mV)	Sheet resistance (Ohms/square)	Resistivity (Ohm.m)	Conductivity (S/cm)
**1:1 PDI-mono(*4*-imz)/PCDDA**	2.26×10^{-3}	0.35	379.8	3.80	$3.12 \times 10^{-3} \pm 0.002$
**2:1 PDI-mono(4-imz)/PDCDDA**	1.05×10^{-3}	0.17	611.12	1.22	$8.48 \times 10^{-3} \pm 0.002$
**1-butyl-3-imidazolium acetate**	N/A	N/A	N/A	N/A	1.44×10^{-3}

Table S11. Ionic conductivity values of **1:1 PDI-mono(*4*-imz)/PCDDA** and **2:1 PDI-mono(4-imz)/PDCDDA** after **UV** exposure in the presence of ionic liquid. The ionic conductivity of 1-butyl-3-imidazolium acetate was reported in the literature.[1]

Sample Name	Applied outer current (mA)	Measured voltage (mV)	Sheet resistance (Ohms/square)	Resistivity (Ohm.cm)	Conductivity (S/cm)
**1:1 PDI-mono(*4*-imz)/PCDDA**	1.29×10^{-5}	1.07	290.0	2.90	$3.448 \times 10^{-4} \pm 0.0001$
**2:1 PDI-mono(4-imz)/PDCDDA**	1.31×10^{-5}	1.09	253.7	2.53	$5.947 \times 10^{-4} \pm 0.0001$
**1-butyl-3-imidazolium acetate**	N/A	N/A	N/A	N/A	1.44×10^{-3}

Table S12. Ionic conductivity values of **1:1 PDI-mono(*4*-imz)/PCDDA** and **2:1 PDI-mono(4-imz)/PDCDDA** after **UV** exposure in the presence of chloroform. The ionic conductivity of 1-butyl-3-imidazolium acetate was reported in the literature.[1]
